# Structural Diversity and Bioactivities of Marine Fungal Terpenoids (2020–2024)

**DOI:** 10.3390/md23080300

**Published:** 2025-07-27

**Authors:** Minghua Jiang, Senhua Chen, Zhibin Zhang, Yiwen Xiao, Du Zhu, Lan Liu

**Affiliations:** 1 Key Laboratory of Natural Microbial Medicine Research of Jiangxi Province, College of Life Sciences, Jiangxi Science and Technology Normal University, Nanchang 330013, China; jiangmh23@mail2.sysu.edu.cn (M.J.); xyw1152858687@163.com (Y.X.); 2 School of Marine Sciences, Sun Yat-Sen University, Zhuhai 519082, China; cesllan@mail.sysu.edu.cn; 3 State Key Laboratory of Environmental Adaptability for Industrial Products, Zhuhai 519082, China; 4 Southern Marine Sciences and Engineering Guangdong Laboratory (Zhuhai), Zhuhai 519082, China; 5 Jiangxi Province Key Laboratory of Biodiversity Conservation and Bioresource Utilization, College of Life Sciences, Jiangxi Normal University, Nanchang 330022, China; zzbbio@jxnu.edu.cn

**Keywords:** terpenoid, biological activity, chemical diversity, marine natural product, marine-derived fungi

## Abstract

Marine-derived fungi have proven to be a rich source of structurally diverse terpenoids with significant pharmacological potential. This systematic review of 119 studies (2020–2024) identifies 512 novel terpenoids, accounting for 87% of the total discoveries to 2020, from five major classes (monoterpenes, sesquiterpenes, diterpenes, sesterterpenes, and triterpenes) isolated from 104 fungal strains across 33 genera. Sesquiterpenoids and diterpenoids constitute the predominant chemical classes, with *Trichoderma*, *Aspergillus*, *Eutypella*, and *Penicillium* being the most productive genera. These fungi were primarily sourced from distinct marine niches, including deep sea sediments, algal associations, mangrove ecosystems, and invertebrate symbioses. Notably, 57% of the 266 tested compounds exhibited diverse biological activities, encompassing anti-inflammatory, antibacterial, antimicroalgal, antifungal, cytotoxic effects, etc. The chemical diversity and biological activities of these marine fungal terpenoids underscore their value as promising lead compounds for pharmaceutical development.

## 1. Introduction

Marine-derived fungi represent a significant source of structurally diverse and pharmacologically active secondary metabolites, notably terpenoids [1,2,3]. Characterized by substantial structural variation, marine fungal terpenoids exhibit broad-spectrum bioactivities, including cytotoxicity, antimicrobial, antiviral, anti-inflammatory, and enzyme inhibitory properties [4,5]. This field experienced unprecedented growth during 2020–2024, aligning with the characterization of this era as the ‘Golden Age of microbial natural product exploration’ (Carroll, A.R. et al.) [6,7]. While prior comprehensive reviews—by Ebel (2011) [8], Elissawy et al. (2015) [9], and Jiang et al. (2020) [10]—tracked a gradual increase in annual terpenoid discoveries from <10 to 61 compounds, the recent surge surpasses historical trends (102 compounds/year).

This exponential increase necessitates an updated systematic review encompassing the structural diversity, bioactivities, and biodiversity of the producing fungi. Our analysis integrates data from 119 primary research articles (2020–2024), reporting 512 newly identified terpenoids derived from marine fungi. Compounds excluded are steroids [11], meroterpenoids (hybrid terpenoids, which constitute a highly active parallel research focus (>460 new compounds reported) [12]), and simple isopentenyl (C_5_)-substituted derivatives.

This rapid expansion underscores the untapped chemical potential of marine fungi and their growing significance in drug discovery. Building on established fungal terpenoid biosynthetic pathways [13], this review provides a contemporary perspective on the fungal taxonomic sources, structural diversity, and biological activities driving progress in marine fungal terpenoid research during 2020–2024.

## 2. The Characteristics of Marine Fungal Terpenoids

**Figure 1:**
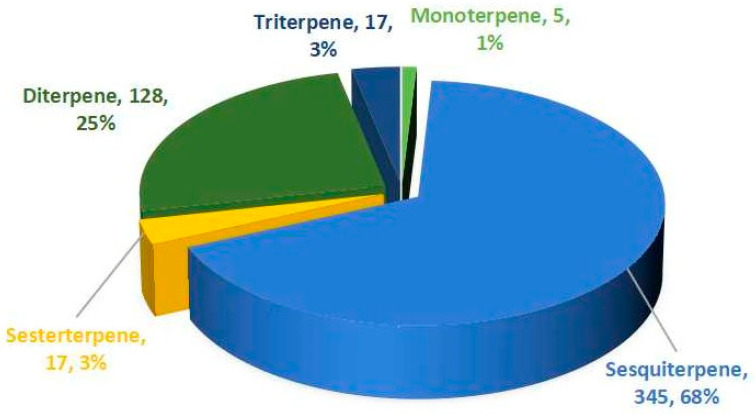
The proportion of different terpenes from marine fungi discovered in 2020–2024.

Analysis of novel terpenoids (C_10_–C_30_) from marine fungi reveals distinct structural groups and a significant surge in discovery rates. Based on chemical structures and biogenetic pathways, these compounds are classified into five groups: mono-, sesqui-, di-, sester-, and triterpenes. Figure 1 depicts the distribution of 512 newly discovered terpenes from marine fungi during 2020–2024. This discovery volume approaches the cumulative total reported prior to 2020 (586 compounds through 2019) [8,9,10], with this accelerated discovery rate likely driven by multiple factors: strategic reallocation of research funding, significant improvements in analytical technologies (e.g., GNPS and NMR), and the systematic implementation of novel approaches (e.g., genome mining) in marine mycology research. Within this new cohort, sesquiterpenes (345 compounds, 68%) and diterpenes (128, 25%) accounted for the vast majority. These were followed by sesterterpenes (17, 3%), triterpenes (17, 3%), and monoterpenes (5, 1%).

**Figure 2:**
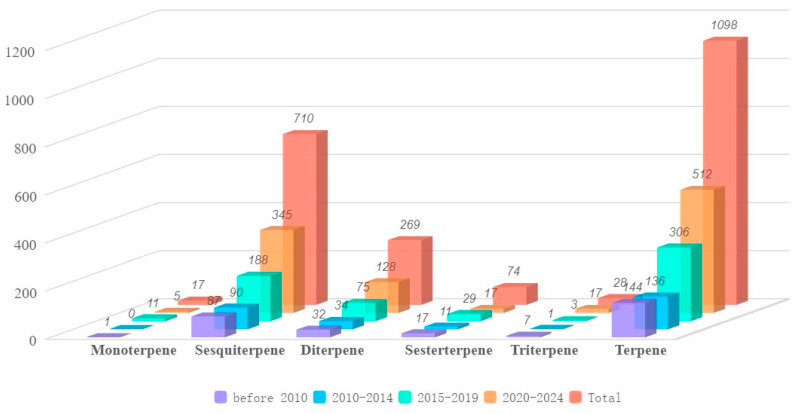
Distribution of novel marine fungal terpenoids as of 2024.

Our comprehensive analysis of the literature data up to 2024 shows that marine fungi are a prolific source of novel terpenoids. A total of 1098 novel terpenoids have been identified from marine fungi to date [8,9,10]. Sesquiterpenes (710, 65%) and diterpenes (269, 24%) remain the predominant classes, together accounting for 979 compounds (89%) of the total (Figure 2). Over the past 15 years, the annual rate of discovery of novel terpenoids from marine fungi has increased dramatically, underscoring its status as a current research hotspot [9,10]. The average annual discovery rate rose sharply from 27 compounds per year between 2010 and 2014 to 61 compounds per year during 2015–2019, reaching 102 compounds per year in 2020–2024 (Figure 2).

**Figure 3:**
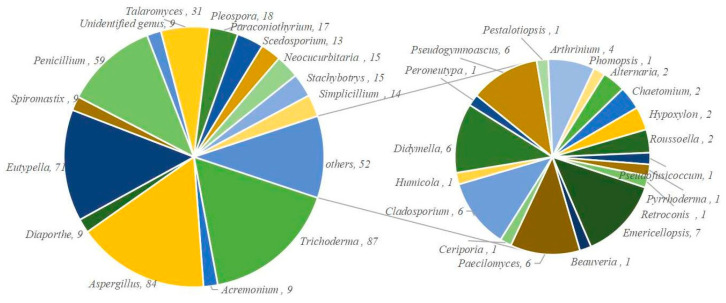
The marine fungal terpenoids divided by the origin of the genera.

In Figure 3, 512 novel terpenes were discovered from 104 strains of diverse marine fungi, spanning over 33 genera (*Trichoderma*, *Aspergillus*, *Eutypella*, *Penicillium*, *Talaromyces*, *Pleospora*, *Paraconiothyrium*, *Neocucurbitaria*, *Stachybotrys*, *Simplicillium*, *Scedosporium (Pseudallescheria)*, *Acremonium*, *Diaporthe*, *Spiromastix*, *Emericellopsis*, *Paecilomyces (Byssochlamys)*, *Cladosporium*, *Didymella* (*Phoma*), *Pseudogymnoascus*, *Arthrinium*, *Alternaria*, *Chaetomium*, *Hypoxylon*, *Roussoella*, *Beauveria*, *Ceriporia*, *Humicola*, *Peroneutypa*, *Pestalotiopsis*, *Phomopsis*, *Pseudofusicoccum*, *Pyrrhoderma*, and *Retroconis*). Four genera each contributed over 10% of the total terpenes and were the dominant producers: *Trichoderma* (17%, 87 compounds), *Aspergillus* (16%, 84), *Eutypella* (14%, 71), and *Penicillium* (12%, 59). Together, these four genera accounted for more than 58% of the total. Seven genera (*Talaromyces*, *Pleospora*, *Paraconiothyrium*, *Neocucurbitaria*, *Stachybotrys*, *Simplicillium*, and *Scedosporium*) produced 2–6% (13–31 compounds each), while the remaining 23 genera collectively contributed 17% (≤9 compounds per genus). Phylogenetic analysis (Figure S1) based on ITS sequences revealed that *Aspergillus*, *Talaromyces*, *Penicillium*, and *Paecilomyces* clustered within a single branch, while *Trichoderma* and *Scedosporium* formed another, and *Eutypella* and *Peroneutypa* grouped together. This suggests that these eight genera are prolific terpene producers, with *Paecilomyces* and *Peroneutypa* showing untapped terpene biosynthesis potential.

**Figure 4:**
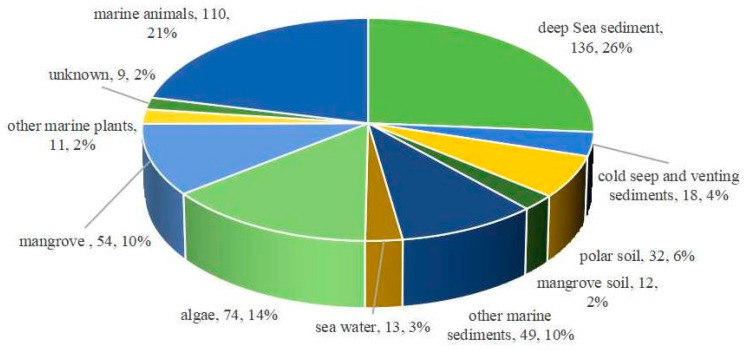
The marine fungal terpenoids were divided by their sources (habitats): 512 terpenoids were isolated from 104 species of fungi in 104 habitats.

Analysis of the habitats and sources of the marine fungi yielding these terpenoids (Figure 4) revealed that the quantity of terpenoid-producing fungi derived from the non-live marine environment (51%, 260) exceeds that of fungi originating from marine organisms (47%, 243). The marine environment comprises deep sea sediments (26%), other marine sediments (10%), etc. The remaining compounds originated from symbiotic associations, specifically aquatic plants (including mangroves and algae, 27%) and marine animals (21%). Among the most significant individual sources (each yielding >30 compounds), deep sea sediments (26%), algae (14%), mangrove habitats (12%), other marine sediments (10%), sponges (7%), polar soil (6%), and coral (6%) were predominant. Notably, extreme environments, e.g., deep sea sediments, cold seep/hydrothermal vent sediments, and polar soil represent emerging and significant sources of marine fungi. Fungi from these habitats are particularly noteworthy for producing structurally unique metabolites.

**Figure 5:**
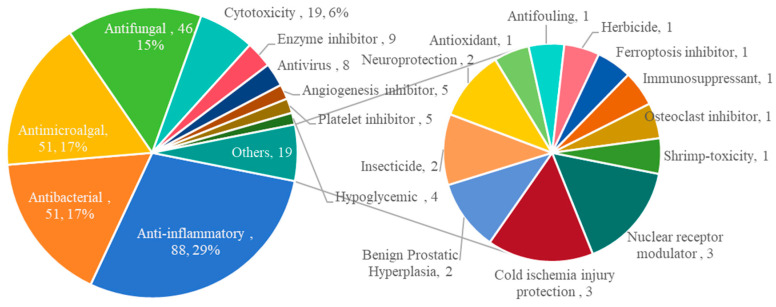
The proportion of biological activities of marine fungal terpenoids (2020–2024).

Naturally occurring terpenoids offer vital drug discovery potential due to their structural diversity and broad bioactivities, exemplified by 512 newly identified fungal terpenoids in this review (Figure 5). Among these, 266 compounds (57% of tested structures) demonstrated one or more biological activities across 305 reports, including 183 sesquiterpenoids, 67 diterpenoids, 7 sesterterpenoids, and 9 triterpenoids. Analysis reveals five predominant activities comprising 83% of the reports: anti-inflammatory (29%, 88 reports), antibacterial (17%, 51), antialgal (17%, 51), antifungal (15%, 46), and cytotoxic (6%, 19). This dominance likely stems from microbial chemical defense mechanisms and common screening bioassays. The remaining 17% covers 17 diverse bioactivities, such as enzyme inhibition, antiviral, antiangiogenic, platelet inhibition, hypoglycemic, nuclear receptor modulation, cold ischemia injury protection, benign prostatic hyperplasia activity, insecticidal, neuroprotective, antioxidant, antifouling, herbicidal, ferroptosis inhibition, osteoclast inhibition, and brine shrimp lethality. These novel marine fungal terpenoids showcase significant structural diversity and multifaceted bioactivities, positioning them as valuable templates for developing therapeutics against diverse diseases.

## 3. Materials and Methods

**Figure 6:**
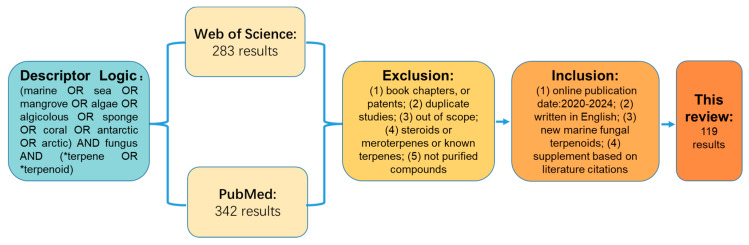
The data collection flowchart of this research.

Data collection for this systematic review utilized the primary online databases Web of Science and PubMed. The search employed the following descriptor logic: (marine OR sea OR mangrove OR algae OR algicolous OR sponge OR coral OR antarctic OR arctic OR polar) AND fungus AND (*terpene OR *terpenoid). The inclusion criteria were (1) online publication date between January 2020 and December 2024, (2) articles published in English, and (3) research articles reporting the first discovery of new terpenes from marine-derived fungi. The initial search identified 629 results (283 from Web of Science and 342 from PubMed). Exclusion criteria applied were (1) book chapters and patents, (2) duplicate studies by Endonote, (3) studies outside the defined scope, (4) studies focusing on steroids or meroterpenes or known terpenes, and (5) compounds that were not purified. Additionally, supplements with data on the discovery of new terpenoids from marine-derived fungi based on literature citations were included. Following this screening process, the systematic review ultimately included 119 publications, reporting 512 compounds (Figure 6).

## 4. Isolation, Structure, and Bioactivities of Marine Fungal Terpenoids

### 4.1. Monoterpenes Five Compounds (**1**–**5**)

**Figure 7:**
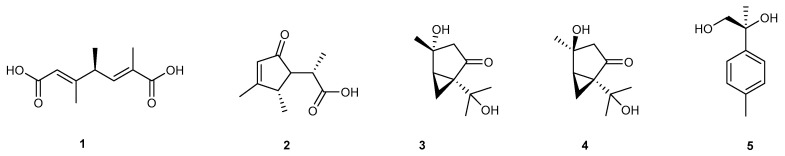
Chemical structures of the monoterpenes (**1**–**5**).

Marine fungi are a notably poor source of monoterpenes; only 16 such compounds have been identified to date, with 5 (**1**–**5**, Figure 7) reported across two articles within the last five years [8,9,10].

Chemical investigation of the culture crude extracts from the deep sea-derived fungus *Aspergillus sydowii* MCCC 3A00324 resulted in the discovery of two osmane-type monoterpenoids, aspermonoterpenoids A and B (**1** and **2**), among which compound **1** was the first osmane with a cleavaged cyclopentane ring. [14] Subsequently, three new monoterpenes, diaporterpenes A–C (**3**–**5**), were obtained from the ascidian-derived fungus *Diaporthe* sp. SYSU–MS4722 [15]. None of the compounds were active when tested for anti-inflammatory activity in vitro.

### 4.2. Sesquiterpenes 345 Compounds (**6**–**350**)

**Figure 8:**
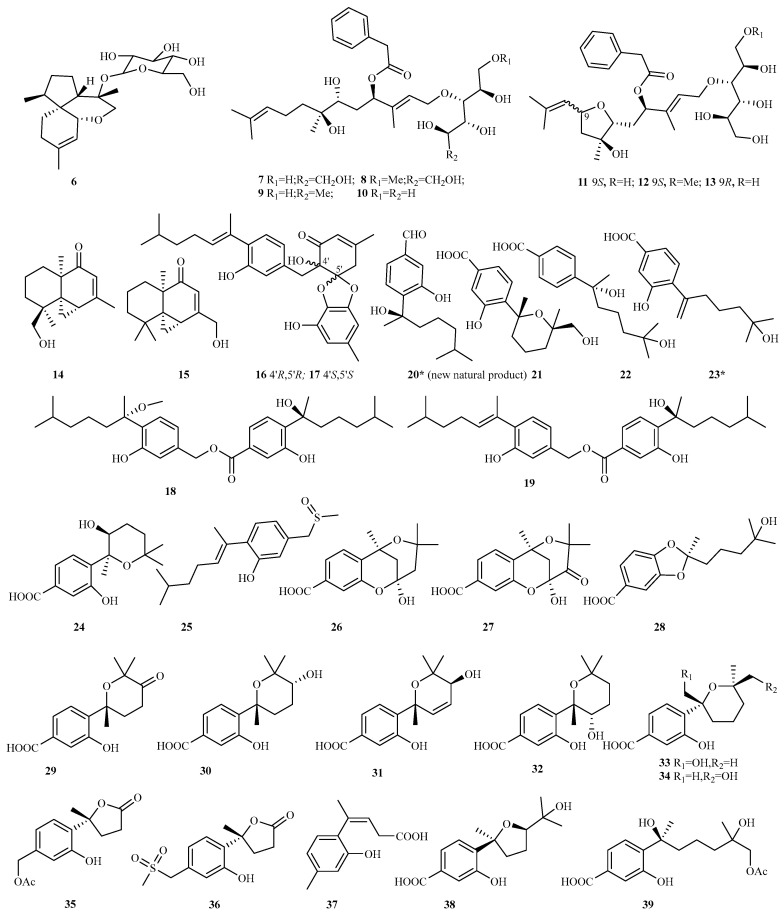
Chemical structures of sesquiterpenes (**6**–**13** from *Acremonium* sp., **14**–**15** from *Alternaria* sp., and **16**–**39** from *Aspergillus* sp.). The * representing new natural products.

**Figure 9:**
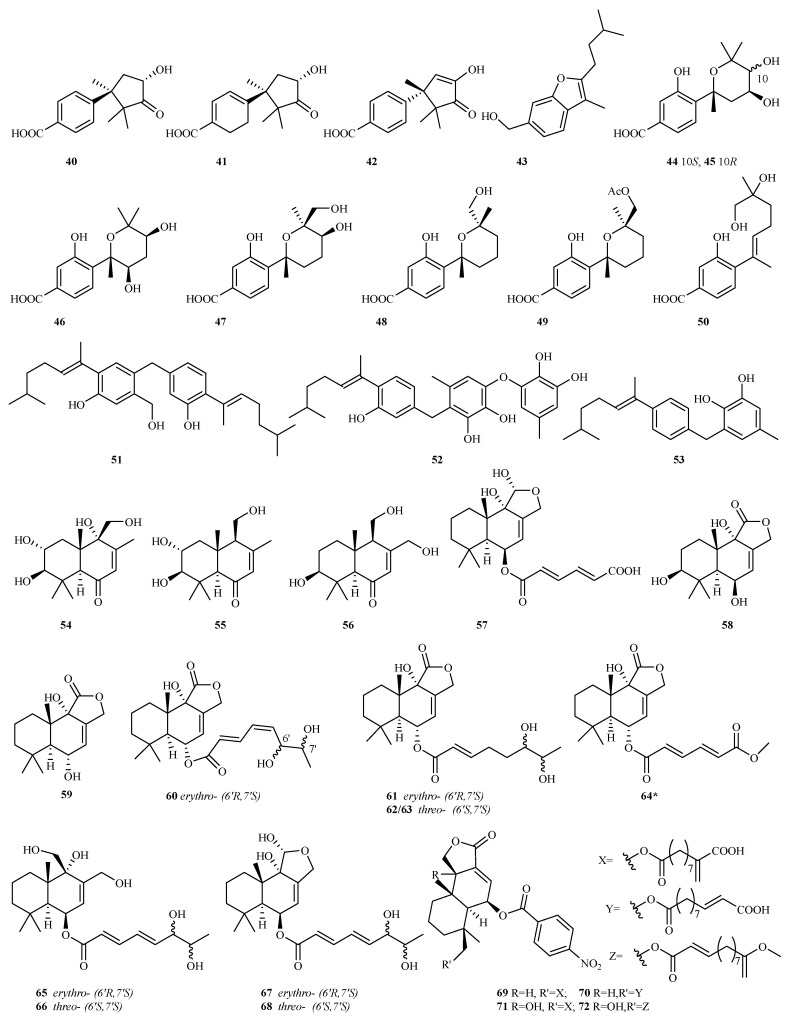
Chemical structures of sesquiterpenes (**40**–**72** from *Aspergillus* sp.). The * representing new natural products.

**Figure 10:**
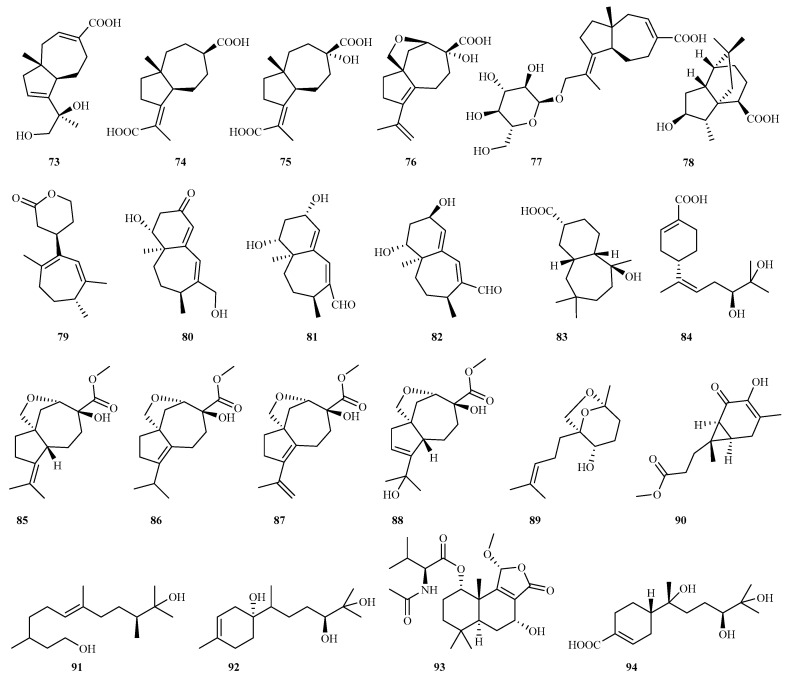
Chemical structures of sesquiterpenes (**73**–**84** from *Aspergillus* sp., **85**–**90** from *Byssochlamys* sp., **91**–**93** from *Cladosporium* sp., and **94** from *Colletotrichum* sp.).

**Figure 11:**
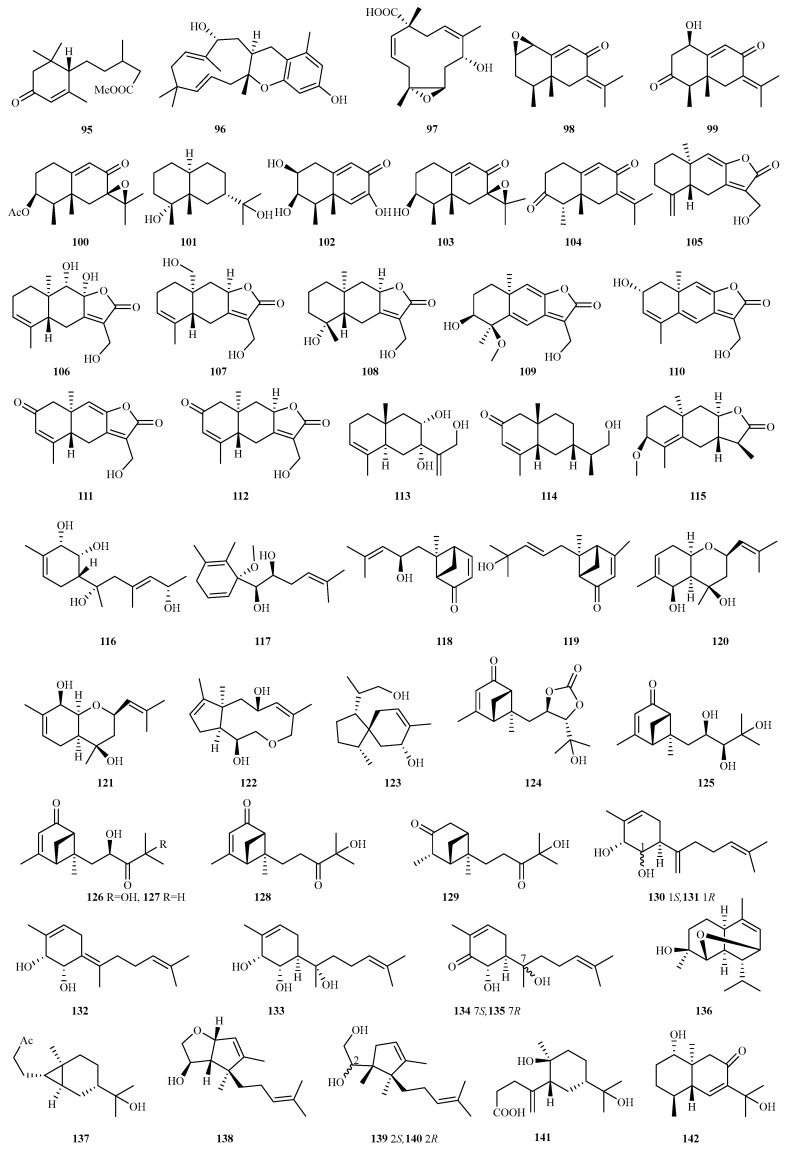
Chemical structures of sesquiterpenes (**95**–**97** from *Diaporthe* sp., **98**–**104** from *Emericellopsis* sp., and **105**–**142** from *Eutypella* sp.).

**Figure 12:**
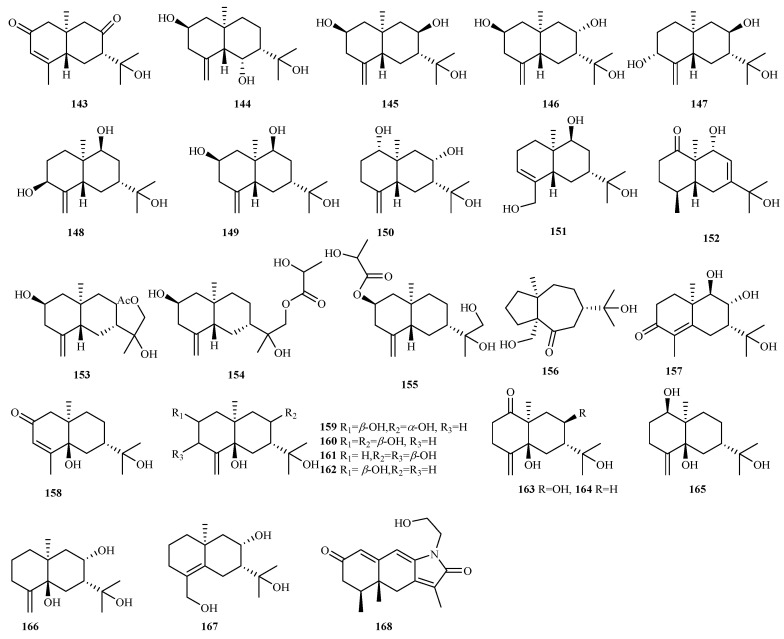
Chemical structures of sesquiterpenes (**143**–**167** from *Eutypella* sp., and **168** from *Humicola* sp.).

**Figure 13:**
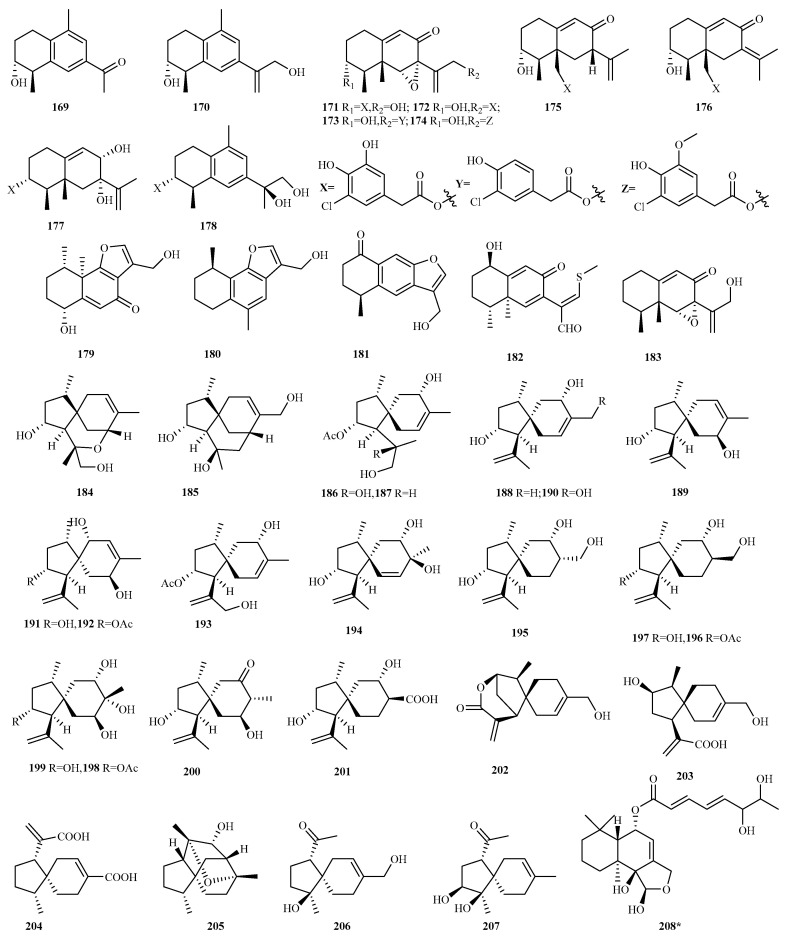
Chemical structures of sesquiterpenes (**169**–**208** from *Penicillium* sp.). The * representing new natural products.

**Figure 14:**
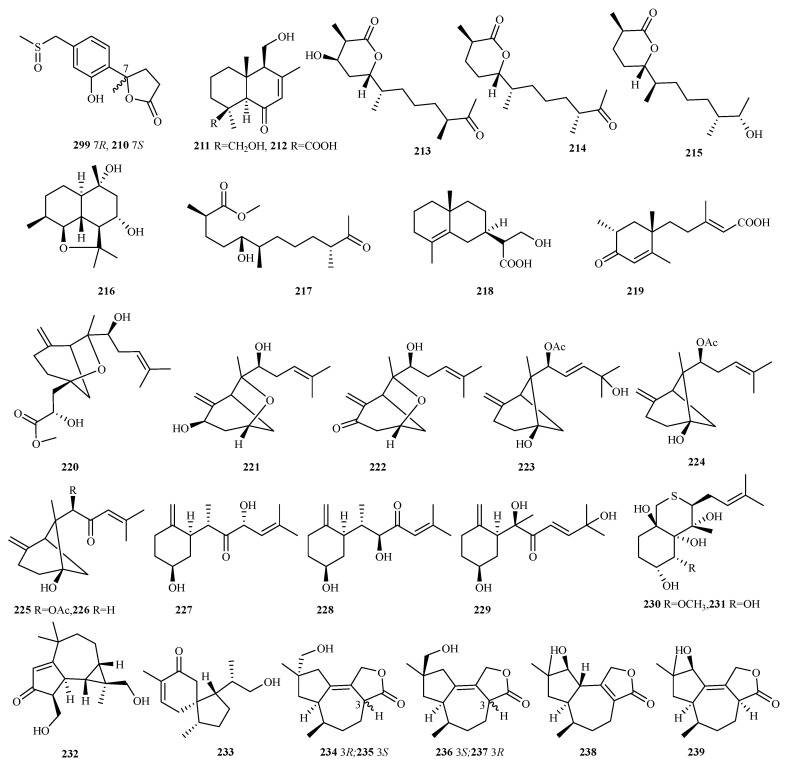
Chemical structures of sesquiterpenes (**209**–**218** from *Penicillium* sp., **219** from *Phoma* sp., **220**–**232** from *Pseudallescheria* sp., **233** from *Pseudofusicoccum* sp., and **234**–**239** from *Pseudogymnoascus* sp.).

**Figure 15:**
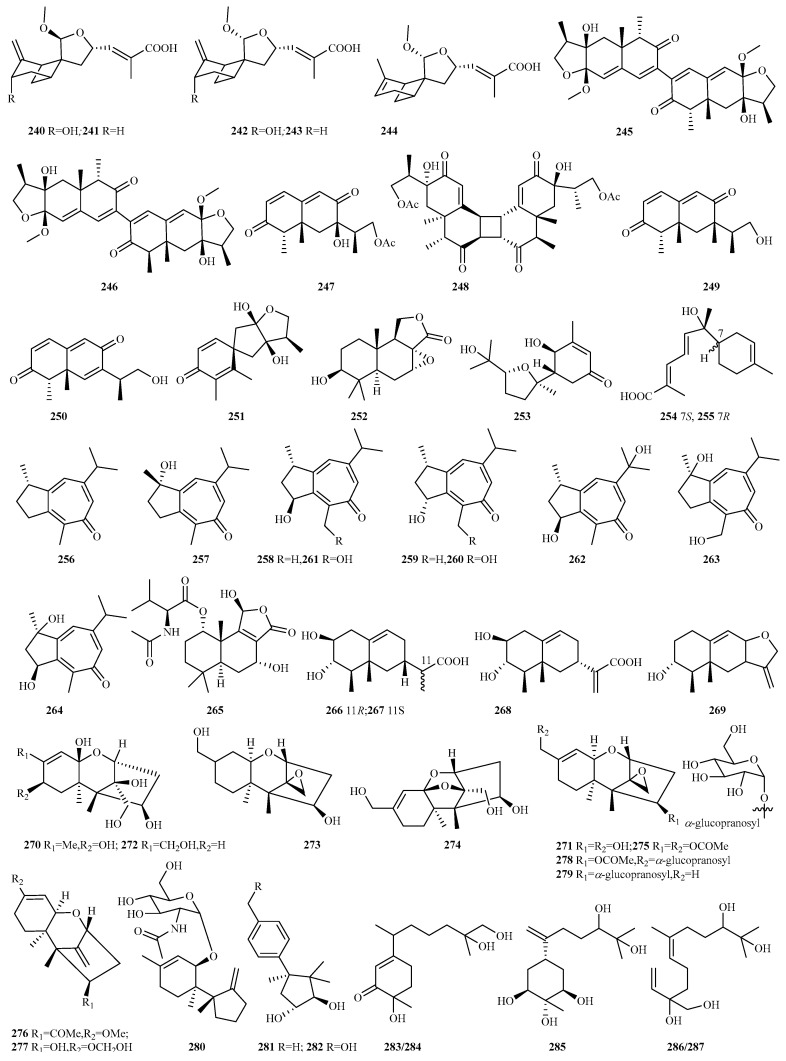
Chemical structures of sesquiterpenes (**240**–**251** from *Paraconiothyrium* sp., **252** from *Pyrrhoderma* sp., **253** from *Retroconis* sp., **254**–**255** from *Roussoella* sp., **256**–**264** from *Spiromastix* sp., and **265**–**287** from *Talaromyces* sp.).

**Figure 16:**
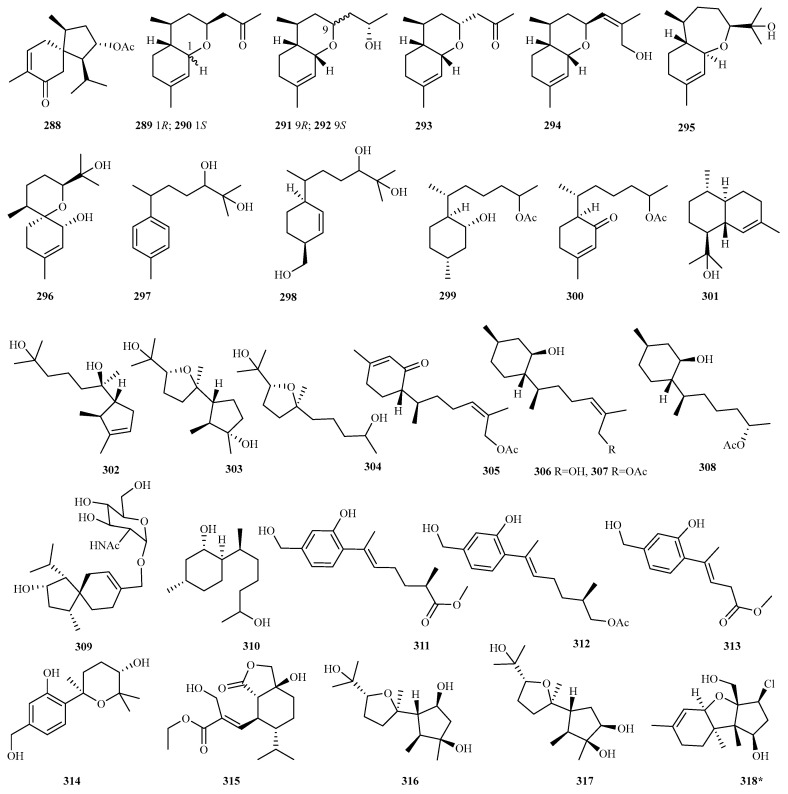
Chemical structures of sesquiterpenes (**288**–**318** from *Trichoderma* sp.). The * representing new natural products.

**Figure 17:**
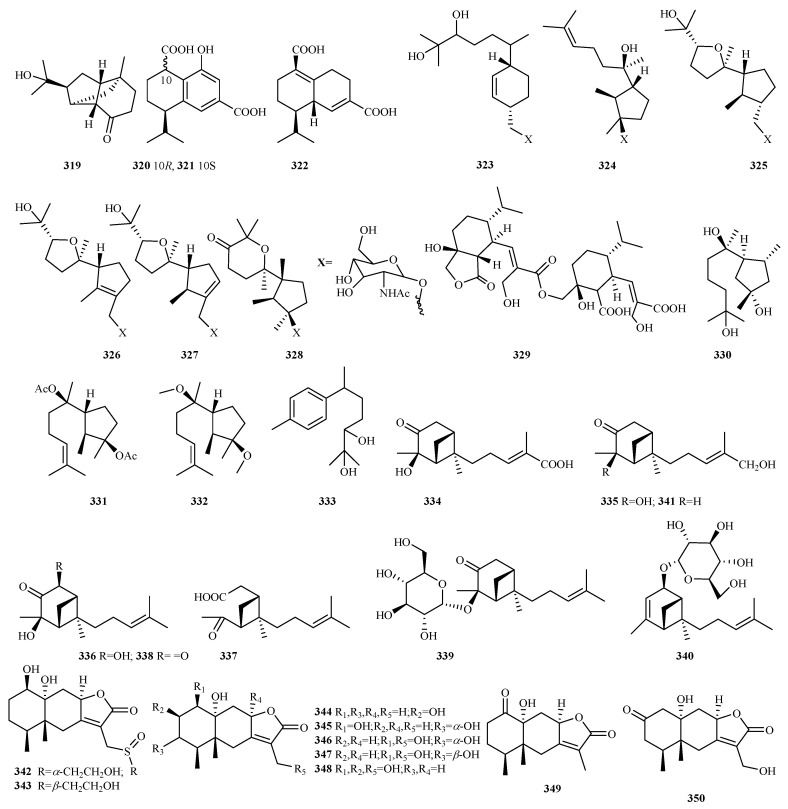
Chemical structures of sesquiterpenes (**319**–**341** from *Trichoderma* sp. and **342**–**350** from *Xylariaceae* sp.).

Sesquiterpenes represent the largest and most prolific group of marine fungal terpenoids. Remarkably, 81 research papers published between 2020 and 2024 describe 345 new sesquiterpenes (**6**–**350**, Figure 8, Figure 9, Figure 10, Figure 11, Figure 12, Figure 13, Figure 14, Figure 15, Figure 16 and Figure 17) from approximately 23 genera. The 345 novel structures discovered in the past five years nearly match the entire pre-2020 cumulative total of 365 compounds [8,9,10], underscoring a dramatically accelerated discovery rate.

The majority of the fungi yielding these new sesquiterpenes were mainly isolated from deep sea sediments (86, 25%); marine algae (65, 19%); mangrove plants (26, 8%); and marine animals, including sponges, coral, and starfish (46, 13%). In terms of fungal genera, the four genera of *Trichoderma* (72, 21%), *Aspergillus* (69, 20%), *Eutypella* (63, 18%), and *Penicillium* (50, 14%) are the major objects of focus by researchers in this field (74% of discoveries), and additional significant producers encompass *Acremonium*, *Alternaria*, *Paecilomyces*, *Cladosporium*, *Colletotrichum*, *Diaporthe*, *Emericellopsis*, *Humicola*, *Didymella*, *Scedosporium*, *Pseudofusicoccum*, *Pseudogymnoascus*, *Paraconiothyrium*, *Pyrrhoderma*, *Retroconis*, *Roussoella*, *Spiromastix*, *Talaromyces*, and one unidentified fungal species. Biologically, among 345 new sesquiterpenoids, 29 remain untested for bioactivity. Of the 316 compounds tested, 183 compounds (58%) exhibit one or more biological activities, corresponding to a total of 207 distinct activity reports. The primary activities (86% of reported activities) comprise anti-inflammatory activity (58 reports, 28%), antimicroalgal activity (44, 21%), antifungal activity (31, 15%), antibacterial activity (27, 13%), and cytotoxicity (17, 8%). The remaining bioactivities (14%) include antiviral effects, enzyme inhibition, antiangiogenic properties, nuclear receptor modulation, neuroprotection, antidiabetic potential, immunosuppression, antioxidant activity, ferroptosis inhibition, antifouling, and herbicidal effects.

#### 4.2.1. *Acremonium* sp. 8 (**6**–**13**)

Chemical investigation of the Antarctic lichen-derived fungus *Acremonium* sp. SF7394 yielded a new acorane-type sesquiterpene glycoside, isocordycepoloside A (**6**) [16]. Subsequently, seven sugar alcohol–conjugated acyclic sesquiterpenes, acremosides A–G (**7–13**), were isolated from the cultures of the sponge-associated fungus *Acremonium* sp. IMB18-086 cultivated with heat-killed pathogenic bacteria *Pseudomonas aeruginosa*. Acremosides E–G possessed a linear sesquiterpene skeleton bearing a tetrahydrofuran moiety linked to a sugar alcohol unit. Bioassays revealed that compounds **7** and **9–11** exhibited significant anti-HCV activity (EC_50_ = 4.8–8.8 μM), whereas compounds **8**, **12**, and **13** displayed moderate efficacy against hepatitis C virus (EC_50_ = 12–16 μM) [17].

#### 4.2.2. *Alternaria* sp. 2 (**14**–**15**)

Two new sesquiterpenoids, alternaterpenoids A and B (**14** and **15**), were isolated from the marine-derived fungus *Alternaria* sp. 5102. Compound **15** exhibited potent anti-inflammatory activity by inhibiting the production of NO in LPS-induced RAW264.7 cells (IC_50_, 18.7 µM) [18].

#### 4.2.3. *Aspergillus* sp. 69 (**16**–**84**)

Aromatic bisabolane and drimane sesquiterpenoids constitute the primary sources of novel sesquiterpenes isolated from marine-derived *Aspergillus* spp. Among these, 38 bisabolane-type compounds (**16**–**53**) have been identified, including 5 rare hetero-/homodimers (**16**–**19** and **51**), as well as 19 drimane-type sesquiterpenoids (**54–72**), highlighting the remarkable chemical diversity of these marine fungal metabolites.

Chemical examination of marine fungus *A. versicolor* A18 led to the isolation of four undescribed hetero-/homodimers of bisabolanes, (+/–)-asperbisabol A **16/17**, and asperbisabols B/C **18**/**19**, together with one new natural monomer, (*R*)-3-hydroxy-4-(2-hydroxy-6-methylheptan-2-yl)benzaldehyde **20**. Within this group, the enantiomers **16** and **17** constitute a rare instance of heterodimers featuring spiroketal diphenyl ether-coupled phenolic bisabolanes. [19] Subsequently, four new bisabolane derivatives (**21**–**22**, and **24** and **25**) have been identified from three marine *Aspergillus* strains. These include 7′-oxygenated sydowic acid (**21)**, (–)-austrosene (**22**), and a natural 3-hydroxy-4-(5-hydroxy-5-methyl-1-methylenehexyyl)-benzoic acid (**23**) from deep sea *Aspergillus* sp. SCSIO06786, [20] (7*S*, 8*S*)-8-hydroxysydowic acid (**24**) from the red alga endophytic *A. sydowii* EN-434 [21], and sulfoxide-containing aspersydosulfoxide A (**25**) from deep sea *A. sydowii* LW09. [22] Among these compounds, compound **16** demonstrated potent neuroprotective activities at a concentration of 10 µM, [19] while compound **24** exhibited significant DPPH free radical scavenging activity with an IC_50_ value of 113.5 μM [21].

Seventeen previously unreported sesquiterpenoids, containing fourteen phenolic bisabolanes, namely asperbisabolanes A–N (**26**–**39**), and three cuparenes (aspercuparenes A–C, **40**–**42**), were discovered from the organic extract of fermented cultures of the deep sea fungus *A. sydowii* MCCC 3A00324. Compounds **26** and **27** represent the first instances of 6/6/6 tricyclic bisabolanes. Compound **28** features a novel *seco*-bisabolane skeleton bearing a rare dioxolane ring group, while **36** was the first example of bisabolanes with an unusual methylsulfonyl moiety. Bioassays showed that compounds **31**, **37**, and **41** exhibited NO inhibition rates exceeding 45% at 10 µM in LPS-activated BV-2 microglia cells. Additionally, compound **37** demonstrated anti-inflammatory properties by inhibiting the NF-*κ*B signaling pathway [23].

Utilizing chemical epigenetic agents at a concentration of 100 mM SAHA and 100 mM 5-Aza in Czapek–Dox medium, a novel bisabolane, named aspergillusene E (**43**), was produced by the gorgonian-derived fungus *A*. *versicolor* XS-20090066. Compound **43** exhibited antibacterial activities against *Staphylococcus epidermidis* and *S. aureus* with MIC values ranging from 8 to 16 μg/mL. It also showed antifungal effects towards *Candida albicans* and *C. tropicalis* with MIC values of 64 and 32 μg/mL, respectively. Furthermore, it demonstrated antifouling properties against bryozoan *Bugula neritina*, with EC_50_ and LC_50_ values of 6.25 and 25 μg/mL, respectively [24].

The deep sea-sourced fungus *A. versicolor* YPH93 yielded seven new phenolic bisabolane sesquiterpenoids (**44**–**50**), among which **44**–**46** represent the first examples of phenolic bisabolanes containing two hydroxy groups attached to the pyran ring. Compound **50** exerted selective inhibition on erastin/RSL3-induced ferroptosis, with EC_50_ values ranging from 2 to 4 μM [25]. Additionally, a rare dimeric bisabolane, aspergol A **51**, and two new monomers, expansol H and aspergol B **52**/**53**, were obtained from the deep sea fungus *Aspergillus* sp. MCCC 3A00392 [26].

Four new drimane sesquiterpenoids (ustusols F–H **54**, **56**, **57**, and 9-deoxyustusol F **55**), along with one known analog, ustusolate I, were isolated from the fermentation broth of the mangrove-associated *A. ustus* 094102. Ustusolate I showed antiproliferative effects against the human tumor cells CAL-62 and MG-63, with IC_50_ values of 16.3 and 10.1 µM, respectively [27].

Six new drimanes, including 3*S*–hydroxystrobilactone A (**58**) and 6-*epi*-strobilactone A (**59**), ustusolate K–N (**60**–**63**), one nature derivate, ustusolate O (**64**), were purified from the cultures of the algicolous *Aspergillus* sp. RR-YLW-12, which is associated with the red alga *Rhodomela confervoides* [28,29]. Compounds **60**–**64** exhibited potential to moderate the inhibition of five microalgae species with IC_50_ values ranging from 5.8 to 54.5 μg/mL [29].

Two previously undescribed drimane sesquiterpenes (ustusolates I, H or asperflavinoids A/B **65**/**66**), were isolated from the seagrass-derived fungus *A. insuetus* SYSU6925. Compounds **65** and **66** demonstrated weak antifungal activities against four phytopathogenic fungi, with MIC values ranging from 100 to 200 μg/mL. Additionally, these compounds exhibited potent anti-inflammatory effects by inhibiting the production of NO in RAW264.7 cells, with IC_50_ values of 32.6 and 21.5 μM, respectively [30]. Concurrently, the chemical analysis of a coculture involving the marine-derived fungi *A. carneus* KMM 4638 and *Beauveria felina* KMM 4639 resulted in the identification of three new drimane derivatives, namely asperflavinoids B, D, and E (**66**–**68**) [31].

Four novel nitrobenzoyl sesquiterpenoids, named insulicolides D–G (**69**–**72**), were extracted from a fungus, *A. insulicola* HDN151418, sourced from an Antarctic sponge. Compounds **71** and **72** demonstrated selective inhibition on human pancreatic ductal adenocarcinoma (PDAC) cell lines (IC_50_, 2.3–4.6 μM). Further research revealed that compound **72** notably suppressed cell proliferation, induced apoptosis, and hindered the migration and invasion of PDAC cells. It also avoided resistance and enhanced the therapeutic efficacy of the chemotherapy drug gemcitabine by inhibiting the expression of EGFR and XIAP in PDAC cells [32].

Five novel carotane sesquiterpenoids (asperalacids A–E **73**–**77**), and a new terrecyclic sesquiterpenoid (4-hydroxy-5(6)dihydroterrecyclic acid A **78**), were isolated from the seagrass-derived *A. alabamensis*. Among these, compound **77** was the first glycosylated carotane sesquiterpenoid with one *α*-D-glucose moiety. Compound **76** exhibited superior inhibitory activity on wheat root and shoot elongation compared to the herbicide terbutryn, identifying it as a potent natural plant growth inhibitor. Compounds **73–76** and **78** also showed weak to potent antimicrobial effects against three plant pathogenic fungi and two bacteria (MIC 25–200 μg/mL) [33].

From the sponge symbiotic fungus *A. niger*, four novel sesquiterpenoids—nigerin (**79**) and ochracenes J–L (**80**–**82**)—were isolated. Nigerin exhibits a rare 1-(3-n-pentyl)-2,5,6-trimethyl-cycloheptane skeleton, while ochracenes J–L represent new humulane-derived derivatives. Nigerin and ochracene J demonstrated a potent inhibition of NO production in LPS-induced RAW264.7 cells, with IC_50_ values of 8.5 and 4.6 μM, respectively [34].

The chemical investigation of rice solid fermentation metabolites from the deep sea-derived fungus *A. puniceus* A2 yielded a new sesquiterpenoid, malfilanol C (**83**). This compound represents the third natural sesquiterpenoid bearing a bicyclo [5.4.0]-undecane nucleus moiety and exerted weak antibacterial effects against *Staphylococcus aureus* [35]. Additionally, gxsespene A (**84**), a novel sesquiterpene isolated from the mangrove endophytic fungus *Aspergillus* sp. GXNU–MA1, demonstrated moderate anti-inflammatory activity against NO production (IC_50_ = 16.15 μM) [36].

#### 4.2.4. *Byssochlamys* (*Paecilomyces*) sp. 6 (**85**–**90**)

Four new carotane sesquiterpenoids, byssocarotins A–D (**85**–**88**), and two new *nor*-sesquiterpenoids, byssofarnesin (**89**) and byssosesquicarin (**90**), were isolated from a macroalga-associated fungus, *Byssochlamys spectabilis* (anamorph *Paecilomyces variotii*) RR-dl-2-13. Compounds **85**–**88** are rare 2,15-epoxycarotane sesquiterpenoids, whereas **89** and **90** represent degradation products of farnesane and sesquicarane precursors, respectively. Compounds **85**–**88** and **90** demonstrated antibacterial activity against marine-derived *Vibrio* spp. [37].

#### 4.2.5. *Cladosporium* sp. 3 (**91**–**93**)

The jellyfish-derived fungus *Cladosporium oxysporum* yielded two novel sesquiterpenes, cladopsol C (**91**) and cladopsol D (**92**) [38]. Meanwhile, the deep sea fungus *Cladosporium* sp. SCSIO 41318 produced a drimane sesquiterpene lactone (purpuride F, **93**) with potent antifungal effects against *Colletotrichum asianum* (MIC = 4 μg/mL) [39].

#### 4.2.6. *Colletotrichum* sp. 1 (**94**)

The mangrove-associated fungus *Colletotrichum* sp. SCSIO KcB3-2 produced a novel bisabolane-type sesquiterpene, bisabolanoic acid A (**94**), which exhibited moderate acetylcholinesterase (AChE) inhibitory activity (IC_50_ = 2.2 μM) [40].

#### 4.2.7. *Diaporthe* sp. 3 (**95**–**97**)

The mangrove endophytic fungus *Diaporthe* sp. SCSIO 41011 yielded a novel sesquiterpenoid, 1-methoxypestabacillin B (**95**), from its solid cultures [41]. From *Diaporthe* sp. QYM12, two new sesquiterpenoids, diaporpenoids B (**96**) and C (**97**), were isolated, showing weak anti-inflammatory effects through NO production inhibition in LPS-induced RAW264.7 cells (IC_50_: 36.8 and 50.0 μM) [42].

#### 4.2.8. *Emericellopsis* sp. 7 (**98**–**104**)

Using the OSMAC approach, the marine sediment-derived fungus *Emericellopsis maritima* BC17 yielded seven new eremophilanes: **98**–**101** from Czapek Dox medium [43] and three compounds **102**–**104** from PDB medium [44]. Notably, compound **104** represents a distinct stereochemical series compared to previously isolate fungal eremophilanes [44].

#### 4.2.9. *Eutypella* sp. 63 (**105**–**167**)

The polar fungus *Eutypella* sp. D-1 yielded 11 novel sesquiterpenes via the OSMAC approach: 8 12,8–eudesmanolides (eutypellaolides A–H, **105**–**112**) and 3 eudesmanes (eutypellaolides I–K, **113**–**115**). Compound **105** showed potent antibacterial activity against *B. subtilis* and *S. aureus* (MIC = 2 μg/mL), while **113** exhibited immunosuppression (ConA-induced T-cell proliferation with 61.7% inhibition at 19.8 μM). Compounds **109** (PTP1B, IC_50_ = 44.8 μM) and **115** (active against *E. coli* and *S. aureus*, MIC = 25 μg/mL) displayed moderate bioactivities [45,46]. Subsequently, gene knockout of triterpene cyclase in this strain induced metabolic shunting, producing eight additional sesquiterpenes (**116**–**123**). Notably, **122** features a rare 5/10 macrocyclic ether scaffold, while **121** and **123** showed anti-inflammatory effects via NO modulation in RAW264.7 cells. The acorane-type **123** additionally suppressed the MAPK and NLRP3/caspase-1 pathways and ameliorated CuSO_4_-induced neuroinflammation in zebrafish [47].

Chemical epigenetic manipulation of deep sea-derived *Eutypella* sp. MCCC 3A00281 yielded 17 novel sesquiterpenes, eutypeterpenes A–Q (**124**–**140**), including bergamotanes (**124**–**129**), bisabolanes (**130**–**135**), cadinene (**136**), carabrane (**137**), and unclassified cyclopentanes (**138**–**140**). Compound **124** represents the first dioxolanone-containing bergamotene. Most compounds, except **109** and **110,** inhibited LPS-induced NO production in RAW264.7 cells (IC_50,_ 8.6–41.5 μM), with **137** showing the strongest activity [48].

Marine-derived *Eutypella* sp. F0219 yielded 15 novel sesquiterpenes, eutypelides A–O (**141**–**155**), including the rare 1,10-*seco-ent*-eudesmane **141** and typical 6/6-fused *ent*-eudesmanes **142**–**155** [49]. Further investigation identified 12 new *ent*-eudesmanes, eutypenes A–L (**156**–**167**), with **156** featuring an unusual 5/7-fused system. Compounds **159**, **161**, **162**, **164**, and **165** dose-dependently inhibited human microvascular endothelial cell line (HMEC-1) cell tube formation. Compound **164** emerged as the most potent and least toxic angiogenesis inhibitor, demonstrating significant tumor antiangiogenic activity in vitro and ex vivo. Mechanistic studies revealed its action through VEGF-A reduction and VEGF-A/VEGFR2 signaling pathway suppression [50].

#### 4.2.10. *Humicola* sp. 1 (**168**)

Chemical analysis of marine-derived *Humicola* sp. GXIMD02070 yielded a new eremophilane sesquiterpenoid (**168**), which displayed moderate anti-inflammatory activity by the inhibition of NO production with an EC_50_ value of 82.04 μM [51].

#### 4.2.11. *Penicillium* sp. 50 (**169**–**218**)

From the sponge-derived fungus *Penicillium copticola* WZXY-m122-9, ten undescribed eremophilanes, copteremophilanes A–J (**169**–**178**), were characterized, featuring rare skeletons with an aromatic ring and methyl migration from C5 to C9 in compounds **169**, **170**, and **178**, alongside chlorinated phenylacetic units in **171**–**177**, a structural motif infrequent in nature. Biological assays demonstrated neuroprotective effects for **175**, selective inhibition of human non-small cell lung cancer A549 cells for **176** (IC_50_, 3.2 µM), and selective inhibition of HCT-8 for **172** and **173** (IC_50_, 5.4 and 7.3 µM) [52].

Mangrove-associated *Penicillium* sp. HDN13-494 produced five novel sesquiterpenoids: citreobenzofurans D–F (**179**–**181**) and phomenones A/B (**182**/**183**). Citreobenzofurans E/F incorporate benzofuran moieties within eremophilane-type frameworks, while phomenone A contains a unique thiomethyl group, marking the first sulfur-containing sesquiterpene. Phomenone B displayed moderate antibacterial efficacy against *Bacillus subtilis* (MIC = 6.25 µM) [53].

Genomic and molecular networking-guided isolation from deep sea *P. bilaiae* F-28 yielded 18 new acorane sesquiterpenes, bilaiaeacorenols A–R (**184**–**201**), characterized by unique tricyclic scaffolds in bilaiaeacorenols A and B. Compound **201** exhibited potent anti-neuroinflammatory activity, dose-dependently suppressing NO production in LPS-induced BV-2 macrophages, inhibiting NF-*κ*B nuclear translocation, and downregulating iNOS and COX-2 expression [54]. Subsequently, another deep sea-derived *P. janthinellum* (SH0301) afforded six acorane-type sesquiterpenes, penijanacoranes A–F (**202**–**207**). Penijanacorane A features a rare lactone within a novel 6/5/6 tricyclic system, whereas penijanacoranes E and F represent the first *nor-*acoranes lacking C1. Notably, penijanacorane C significantly inhibited LPS-induced NO production in Raw264.7 macrophages (IC_50_ = 6.23 μM), outperforming dexamethasone (IC_50_ = 11.49 μM), and enijanacorane D displayed moderate antivirus against H1N1, with an inhibitory rate of 52.9% at 25 μM [55].

The marine red alga-derived endophytic fungus *P. chrysogenum* LD-201810 yielded chrysoride A (**208**), a novel natural drimane sesquiterpene ester that demonstrated moderate cytotoxicity against HepG2 (IC_50_ = 28.9 µM) and HeLa (IC_50_ = 35.6 µM) cell lines [56]. Subsequent investigation of the same fungal strain led to the isolation of (+/−)-methylsulfinyl-1-hydroxyboivinianin A **209/210**, a pair of aromatic *nor*-bisabolane derivative enantiomers featuring the rare natural methylsulfinyl substituent [57].

From the hydrothermal vent sediment-derived *Penicillium* sp. TW58-16, researchers obtained two new drimane sesquiterpenes (**211** and **212**), with compound **211** showing moderate inhibition of iNOS expression and significant *α*-glucosidase inhibitory activity (35.4%) comparable to, or better than, acarbose [58].

Three novel sesquiterpenoids, chermesiterpenoids A–C (**213**–**215**), were isolated from red alga *Pterocladiella tenuis*-derived *P. chermesinum* EN-480, among which compounds **214** and **215** exhibited broad-spectrum antimicrobial activity against human pathogens, aquatic bacteria, and plant pathogenic fungi (MIC, 0.5–64 µg/mL) [59].

Mangrove-associated *P. oxalicum* KMM 4683 produced (3*β*, 4*β*, 5*β*, 6*β*, 7*β*, 9*β*, and 10*α*)-4,6-epoxy-7-hydroxy-9-cadinanol (**216**), a new cadinane-type sesquiterpene with a unique stereochemical configuration [60].

Chemical analysis of *P. rubens* AS-130 from the deep sea Magellan Seamount resulted in the identification of chermesiterpenoid D (**217**), a structurally distinct linear sesquiterpenoid [61]. Fermentation of mangrove rhizosphere soil-derived *Penicillium* sp. HK1-22 generated artemihedinic acid N (**218**), representing a new addition to the eudesmane-type sesquiterpenoid family [62].

#### 4.2.12. *Phoma (Didymella)* sp. 1 (**219**)

Chemical investigation of the deep sea sulfide-derived fungus *Phoma (Didymella)* sp. 3A00413 led to the isolation of a novel sesquiterpenoid (**219**) [63].

#### 4.2.13. *Pseudallescheria* (*Scedosporium*) sp. 13 (**220**–**232**)

A systematic investigation of the cold seep sediment-derived fungus *Pseudallescheria boydii* CS-793 (the sexual morph of the *Scedosporium* species) yielded ten novel bergamotene-type sesquiterpenoids, pseuboyenes A–J (**220–229**). Compound **220** represents the first *β*-bergamotene featuring a 6-oxobicyclo[3.2.1]octane nucleus adduct with a methyl lactate unit, while **227**–**229** exhibit a skeletal rearrangement from the bergamotene scaffold. Compounds **221**–**226** demonstrated significant antifungal activity against 11 plant pathogens (MICs = 0.5–32 μg/mL). Notably, **223** exhibited excellent antifungal effects towards *Fusarium proliferatum*, *Alternaria Berk.* Sacc, and *Colletotrichum diplodiella* (MIC = 0.5 μg/mL), and **228** showed potent antibacterial activity against *Vibro vulnificus* (MIC, 2 μg/mL) [64]. Additionally, pseudallenes A and B (**230** and **231**), novel sulfur-containing ovalicin derivatives and rare structural examples, were characterized from this strain and showed broad-spectrum inhibitory activities against several plant pathogenic fungi (MIC, 2–16 μg/mL) [65]. Furthermore, a new aromadendrane sesquiterpenoid, pseuboydone F (**232**), was isolated from soft coral *Sarcophyton* sp.-associated fungus *P. boydii* F44-1 [66].

#### 4.2.14. *Pseudofusicoccum* sp. 1 (**233**)

Acorenone C **233**, a new spiro-sesquiterpene from a mangrove-associated fungus, Pseudofusicoccum sp. J003, showed mild AChE inhibitory activity [67].

#### 4.2.15. *Pseudogymnoascus* sp. 6 (**234**–**239**)

Chemical investigation of secondary metabolites from the Antarctic psychrophilic pathogenic fungus *Pseudogymnoascus* sp. HSX2#–11 resulted in the isolation of six novel tremulane-type sesquiterpenoids, designated as pseudotremulanes A–F (**234**–**239**). Structural elucidation revealed that pseudotremulanes A–F constitute a series of structural isomers sharing an identical molecular formula [68].

#### 4.2.16. *Paraconiothyrium* sp. 12 (**240**–**251**)

Five novel bergamotane-type sesquiterpenoids, brasilterpenes A–E (**240**–**244**), featuring a previously unreported 6/4/5 spiral tricyclic ring system, were isolated from the deep sea-derived ascomycete fungus *Paraconiothyrium brasiliense* HDN15-135. Compounds **240** and **242** significantly lowered the blood glucose levels in hyperglycemic zebrafish in vivo by enhancing insulin sensitivity and inhibiting gluconeogenesis. Notably, the hypoglycemic efficacy of compound **242** paralleled that of the antidiabetic drug rosiglitazone (positive control), indicating **242** possesses promising antidiabetic potential [69].

Separately, seven new eremophilane sesquiterpenoids, paraconulones A–G (**245**–**251**), were purified from the ethyl acetate extract of the marine mud-derived fungus *P. sporulosum* DL-16. Compounds **245**, **246**, and **248** represent the first microbial-derived dimeric eremophilane sesquiterpenoids linked via a C–C bond. Compounds **246**–**249** and **251** exhibited inhibitory effects on LPS-induced NO production in BV2 microglial cells (IC_50_ = 2.8–8.1 µM), with potency comparable to the positive control curcumin [70].

#### 4.2.17. *Pyrrhoderma* sp. 1 (**252**)

Phytochemical analysis of the fermentation broth from the marine sediment-derived fungus *Pyrrhoderma noxium* HNNU0524 led to the isolation of a novel drimane-type sesquiterpenoid, designated pyrrnoxin A (**252**) [71].

#### 4.2.18. *Retroconis* sp. 1 (**253**)

The newly identified sesquiterpenoid retrobisabolane A (**253**) was isolated from fermented cultures of the deep sea-derived fungus *Retroconis fusiformis* MCCC 3A00792. Compound **253** represents a structurally unique variant within the bisabolane family, characterized by an unusual methyl group migration from the conventional C_3_ position to the C_4_ position of the sesquiterpenoid skeleton [72].

#### 4.2.19. *Roussoella* sp. 2 (**254** and **255**)

Two novel sesquiterpenoids, elgonenes M (**254**) and N (**255**), were isolated from the mangrove sediment-derived fungus *Roussoella* sp. SCSIO 41427. Elgonene M showed 31.14% inhibition of pro-inflammatory IL-1*β* mRNA at 5 µM, while elgonene N exhibited 27.57% inhibition at 20 µM [73].

#### 4.2.20. *Spiromastix* sp. 9 (**256**–**264**)

SAHA-induced epigenetic modulation of the deep sea *Spiromastix* sp. activated a terpene biosynthetic cluster, producing nine novel guaiane-type sesquiterpenes with rare tropone moieties, spiromaterpenes A–I (**256**–**264**). Among these, compounds **259**–**261** significantly inhibited NO production in LPS-stimulated BV2 microglia (IC_50_ = 9–26 µM). Lead compound **260** exerted anti-inflammatory effects via the dual inhibition of NF-*κ*B signaling and downstream mediators (iNOS/COX-2), with SAR highlighting the critical 2(*R*),11-diol pharmacophore [74].

#### 4.2.21. *Talaromyces* sp. 5 (**265**–**269**)

From deep sea cold seep sediments of the South China Sea, *Talaromyces minioluteus* CS-113 yielded a novel drimane lactone, 11-hydroxyminioluteumide B (**265**). [75] Separately, mangrove-derived *Talaromyces* sp. SCSIO 41412 produced four new sesquiterpenoids, talaroterpenes A–D (**266**–**269**). At 200 µM, compounds **266**, **268**, and **269** activated ABCA1 and PPAR*α*, with **269** showing the greatest potency. Compound **269** also modulated ROR*α* signaling by altering *CLOCK*/*BMAL*1 expression, emerging as a promising non-toxic nuclear receptor modulator [76].

#### 4.2.22. *Trichoderma* sp. 72 (**270**–**341**)

Marine-derived *Trichoderma brevicompactum* NTU439 (isolated from alga *Mastophora rosea*) yielded four new trichothecenes (**270–273**) exhibiting a potent inhibition of LPS-induced NO production (IC_50_, 8.1–12.4 μM) [77]. Subsequent investigation of *T. brevicompactum* A-DL-9-2 (host: red alga *Chondria tenuissima*) discovered eight new trichothecenes (trichodermarins G–N; **271** and **274**–**280**) and two novel cuparene sesquiterpenes (trichocuparins A/B, **281**/**282**). Notably, **280** represents the first trichothecene incorporating an aminosugar moiety, while **281** and **282** constitute the inaugural report of cuparenes within the *Trichoderma* genus. Several trichodermarins (G–I and L–M) displayed inhibitory activity against fungi and phytoplankton (MIC, 32–64 μg/mL; IC_50_, 13–66 μg/mL) [78]. This same fungal strain also produced six new sesquiterpenoids: three bisabolanes (trichobisabolins O1, O2, and P; **283**–**285**), two nerolidols (trichonerolins A/B; **286**/**287**), and one acorane (trichoacorin A; **288**). Among these, **285** and **286/287** potently inhibited the marine phytoplankton *Amphidinium carterae* (IC_50_, 1.8 μg/mL) and *Chattonella marina* (IC_50_, 1.2 μg/mL) [79].

Further exploration of two algicolous *Trichoderma* species yielded 16 novel sesquiterpenes. *T. atroviride* RR-dl-3-9 provided the bisabolanes trichobisabolins M (**289**) and N (**290**) [80], while *T. asperelloides* RR-dl-6-11 produced ten bisabolanes (trichobisabolins Q–Z; **291**–**300**), one cadinane (cadin-4-en-11-ol; **301**), and three cycloneranes (cycloner-3-en-7,11-diol, **302**; isoepicyclonerodiol oxide, **303**; norepicyclonerodiol oxide, **304**) [81]. Significantly, half of these bisabolanes possess only 14 carbon atoms. Compound **302** is the first cyclopentenyl-bearing cyclonerane, and **304** is postulated to arise from degradation of the typical cyclonerane skeleton. These isolates (**291**–**304**) exhibited inhibition against four marine phytoplankton species (IC_50_ 0.54–11 μg/mL) [81].

Additionally, endophytic fungi from marine red alga yielded two types of novel sesquiterpenoids. From *T. asperellum* EN-764, four previously undescribed bisabolane-type sesquiterpenoids (**305**–**308**, (*Z*)-12–acetoxybisabol-1-one, bisabolen-1,12-diol, 12-acetoxybisabolen-1-ol, and 12-*nor-*11-acetoxybisabolan-1-ol) were isolated, exhibiting inhibitory activity against aquatic pathogenic bacteria (MIC: 4–64 μg/mL) [82]. Meanwhile, *T. longibrachiatum* EN-586 yielded trichoacorside A (**309**), representing the first reported glucosamine-conjugated acorane-type sesquiterpenoid. The molecule **309** exhibited moderate activity against methicillin-resistant *Staphylococcus aureus*, the aquatic pathogenic bacterium *Vibrio harveyi*, and several plant-pathogenic fungi (MIC, 4–64 µg/mL) [83].

*Nor-*bisabolan-1,11-diol (**310**) was isolated from marine sediment-derived *T. atroviride* TD–8. It exhibited moderate cytotoxicity against human cancer cell lines (HeLa, IC_50_ = 28.6 μM; HCT-8, IC_50_ = 30.3 μM) [84].

Trichoderenes A–D (**311**–**314**), four novel sesquiterpenes, were obtained via bioassay- and HPLC-guided isolation from marine *T. effusum* HBU-2019-190. Trichoderene C possesses a unique C_12_ norsesquiterpene skeleton. Trichoderenes A–C inhibited bacteria *Agrobacterium tumefaciens* growth (MIC, 3.1–12.5 μg/mL) [85].

Ethyl hydroheptelidate (**315**) from mangrove endophytic *T. harzianum* R1 demonstrated high antifungal activity against plant pathogens *Fusarium oxysporum* and *Colletotrichum musae*, alongside moderate-to-weak antibacterial activity against avian pathogenic *Escherichia coli* strains [86].

Cyclonerane sesquiterpenes (5-hydroxyepicyclonerodiol oxide **316** and 4-hydroxyepicyclonerodiol oxide **317**) and trichodermol chlorohydrin (**318**), a natural halogenated trichothecane, were isolated from the epiphytic *T. hamatum* Z36-7 associated with red alga *Grateloupia* sp. Compound **317** features an unusual 4-hydroxy group on its five-membered ring, while **318** represents the first naturally occurring halogenated trichothecane. These compounds inhibited growth in several bacteria and phytoplankton species [87].

Norpupukeanane A (**319**), a rare norsesquiterpene characterized by an unprecedented tricyclic-6/5/5-[4.3.1.0^1,6^]decane skeleton, was isolated from marine-derived *T. longibrachiatum* (host: halophyte *Suaeda glauca*). It exhibited potent antifungal activity against *Colletotrichum lagrnarium* (MIC = 8 μg/mL; superior to carbendazim, MIC = 32 μg/mL) and efficacy against carbendazim-resistant *Botrytis cinerea* [88].

Three new cadinane sesquiterpenes, trichodermaloids A–C (**320**–**322**), were discovered from sponge-derived *Trichoderma* sp. SM16 [89]. Trichaspside F (**323**) and cyclonerosides A–E (**324**–**328**), six new sesquiterpene aminoglycosides, were isolated from the deep sea sediment-derived *Trichoderma* sp. SCSIOW21. Cyclonerosides A–E represent the first glycosides of cyclonerane-type sesquiterpenes in *Trichoderma* [90]. Additionally, a new sesquiterpene dimer, divirensol H (**329**), was obtained from sponge-derived *T. virens* CMB-TN16 [91]. Bioassays indicated that compounds **320**–**322** and **329** displayed cytotoxicity against human cancer cell lines (NCI-H460 lung, NCI-H929 myeloma, and SW620 colorectal; IC_50_ = 6.8–13.5 μM) [89,91]. Compounds **323** and **325**–**328** exhibited potent NO production inhibitory activities (IC_50_, 42.0–57.1 µM), comparable to quercetin (IC_50_, 30.8 µM) [90].

Chemical epigenetic manipulation (10 µM sodium butyrate) of soft coral-derived *T. harzianum* XS-20090075 yielded 3,7,11-trihydroxy-cycloneran (**330**), a new cyclonerane sesquiterpenoid [92].

Cyclonerodiols A/B (**331**/**332**) and trichodermaerin A (**333**) were isolated from the marine-derived *T. erinaceum* F1-1 cultured in GPY medium without L-phenylalanine [93]. Genome mining of this fungal strain enabled heterologous expression of a silent *bgt* terpene cluster, discovering eight undocumented bergamotene-derived sesquiterpenoids, oxybergamotenes A–H (**334**–**341**) [94].

#### 4.2.23. Unidentified Fungal Species 9 (**342**–**350**)

Based on the chemical investigation of large-scale fermentation products from the mangrove-derived xylariaceous fungus TBRC-BCC 64093 (unidentified species), two unusual eremophilanolide sulfoxide diastereomers (**342** and **343**) and seven new eremophilanolides (**344**–**350**) were isolated and characterized. Compound **342** exhibited weak cytotoxic activity against Vero cells, with an IC_50_ value of 17.7 μg/mL [95].

### 4.3. Diterpenes 128 Compounds (**351**–**478**)

**Figure 18:**
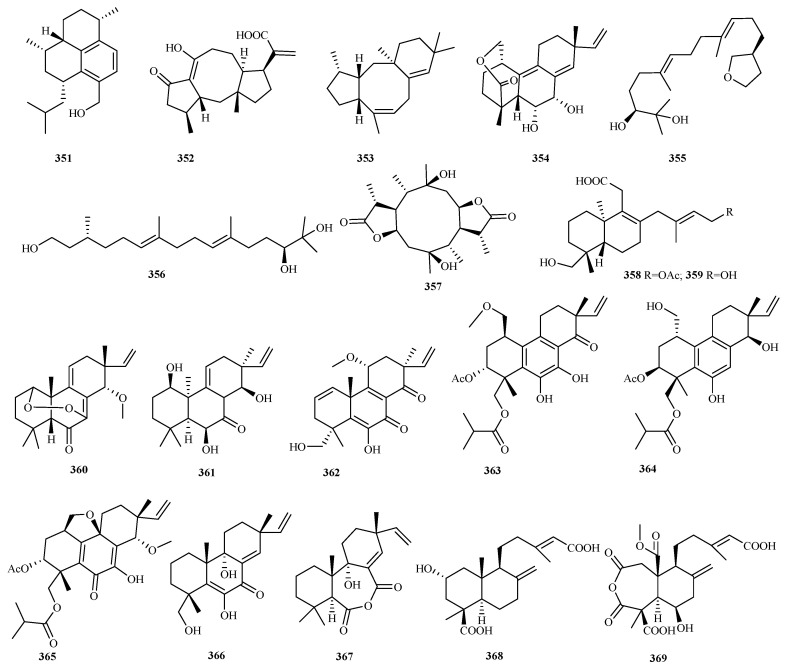
Chemical structures of diterpenes (**351** from *Acremonium* sp., **352**–**353** from *Aspergillus* sp., **354** from *Beauveria* sp., **355**–**356** from *Cladosporium* sp., **357**–**359** from *Diaporthe* sp., **360**–**367** from *Eutypella* sp., and **368**–**369** from *Hypoxylon* sp.).

**Figure 19:**
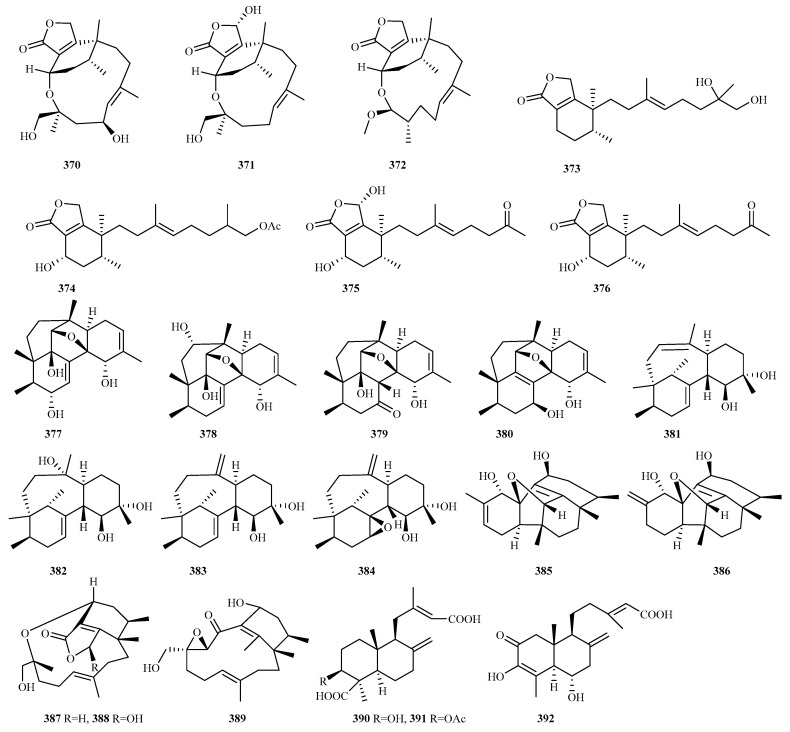
Chemical structures of diterpenes (**370**–**384** from *Neocucurbitaria* sp., **385**–**389** from *Paraconiothyrium* sp., and **390**–**392** from *Penicillium* sp.).

**Figure 20:**
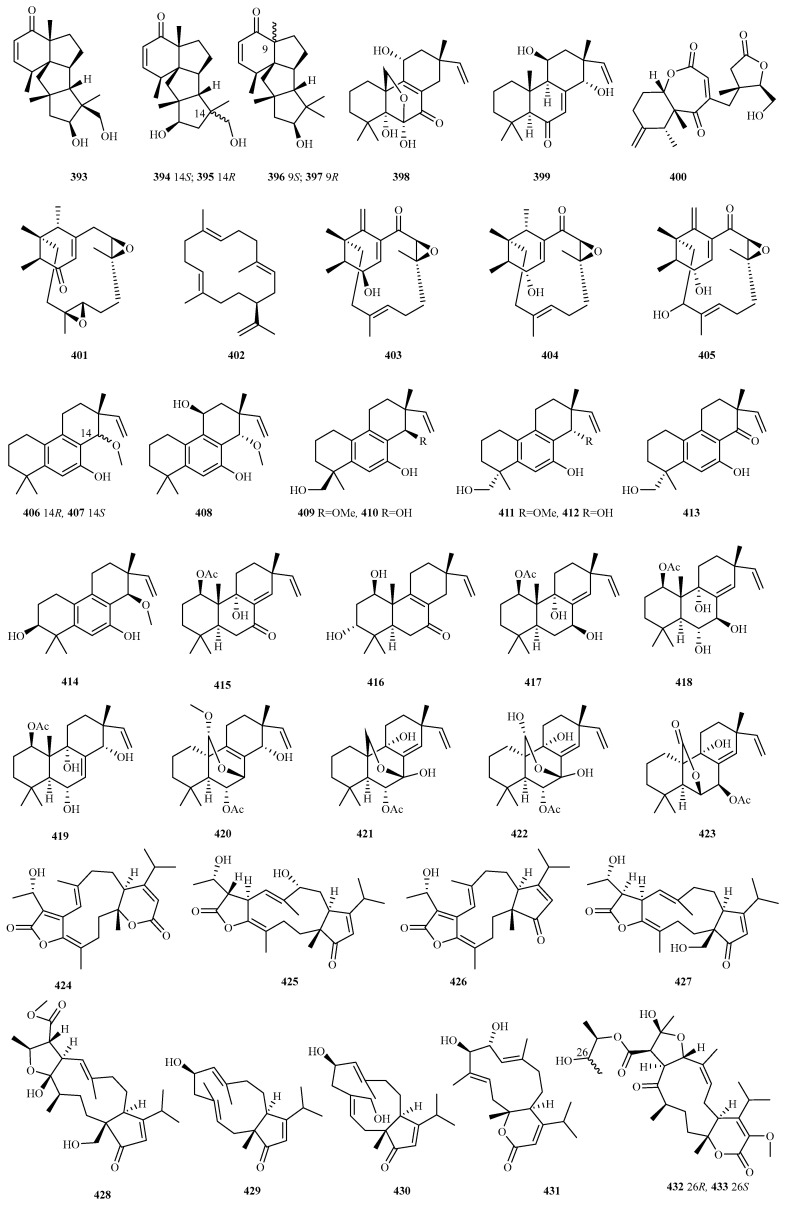
Chemical structures of diterpenes (**393**–**398** from *Penicillium* sp., **399** from *Peroneutypa* sp., **400** from *Pestalotiopsis* sp., **401**–**405** from *Phoma* sp., **406**–**423** from *Pleospora* sp., and **424**–**433** from *Stachybotrys* sp.).

**Figure 21:**
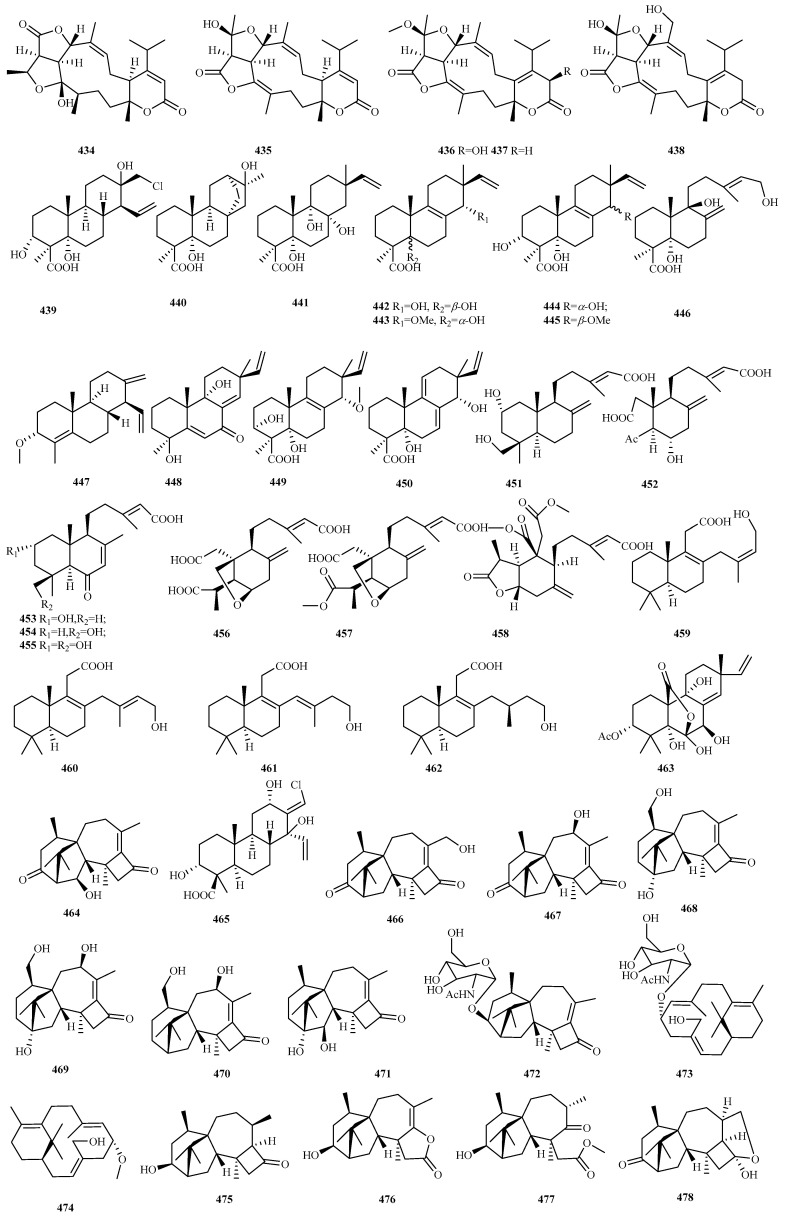
Chemical structures of diterpenes (**434**–**438** from *Stachybotrys* sp., **439**–**463** from *Talaromyces* sp., and **464**–**478** from *Trichoderma* sp.).

Marine fungal diterpenoids constitute a structurally diverse class of terpenes with significant bioactivities [96]. Recent investigations (2020–2024) documented 128 novel diterpenes (**351**–**478**, Figure 18, Figure 19, Figure 20, Figure 21) from marine fungi across 36 studies. This five-year discovery output approaches the cumulative pre-2020 total (141 compounds) [8,9,10], demonstrating the accelerated identification of fungal diterpenes.

The newly reported diterpenes were isolated from marine fungi inhabiting diverse ecological niches, with the highest yield originating from fungi associated with marine animals (including deep sea fauna; 42 compounds, 33%), followed by deep sea sediments (33, 26%), mangrove plants (27, 19%), and polar environments (8, 6%). Taxonomic analysis revealed these producers span 17 fungal genera, with five dominant groups each contributing over 10% of the total: *Talaromyces* (25, 20%), *Pleospora* (18, 14%), *Neocucurbitaria* (15, 12%), *Stachybotrys* (15, 12%), and *Trichoderma* (15, 12%). Minor contributors included *Acremonium*, *Aspergillus*, *Beauveria*, *Cladosporium*, *Diaporthe*, *Eutypella*, *Hypoxylon*, *Paraconiothyrium*, *Penicillium*, *Peroneutypa*, *Pestalotiopsis*, and *Didymella*, all yielding comparatively fewer compounds. Biological evaluation of the new diterpenes revealed that 56% (67 out of 120 tested compounds) exhibited discernible bioactivity. The predominant activities included anti-inflammatory (36%), antifungal (20%), and antibacterial (17.5%) properties, with additional observed effects encompassing antimicroalgal activity, platelet inhibition, and enzyme inhibition, among others.

#### 4.3.1. *Acremonium* sp. 1 (**351**)

Chemical investigation of the Antarctic lichen-derived fungus *Acremonium* sp. SF7394 yielded acrepseudoterin (**351**), a novel amphilectane-type diterpene. This compound exhibited dose-dependent inhibition of protein tyrosine phosphatase 1B (PTP1B) activity, with an IC_50_ value of 22.8 μM [16].

#### 4.3.2. *Aspergillus* sp. 2 (**352**–**353**)

Aculeaterpene A (**352**) was characterized as the first fusicoccane-type norditerpenoid featuring C_20_ degradation with concomitant oxidation to a hydroxy group, isolated from the marine-derived fungus *Aspergillus aculeatinus* WHUF019813 [97]. Heterologous expression of a cryptic bifunctional diterpene synthase *Tnd*C from *A. flavipes* CNL-338 yielded talarodiene (**353**), a novel diterpene scaffold containing a benzo[*a*]cyclopenta[*d*]cyclooctane tricyclic-fused ring system [98].

#### 4.3.3. *Beauveria* sp. 1 (**354**)

The marine bryozoan-derived fungus *Beauveria felina* EN-135 yielded felinane B (**354**), a novel tricyclic diterpenoid. Notably, **354** demonstrated potent antifungal activity against carbendazim-resistant *Botrytis cinerea*, exhibiting MIC values of 32 μg/mL, over carbendazim (MIC = 256 μg/mL) [99].

#### 4.3.4. *Cladosporium* sp. 2 (**355**–**356**)

Two novel acyclic diterpenoids, cladopsol A (**355**) and B (**356**), were isolated from the jellyfish-derived fungus *Cladosporium oxysporum*. Compound **356** exhibited peroxisome proliferator activated receptor-*γ* (PPAR-*γ*) partial agonist in luciferase assays and docking studies, suggesting antidiabetic potential with reduced side effects versus full agonists. Notably, **356** enhanced the glucose uptake in HepG2 cells comparably to rosiglitazone while causing significantly less lipid accumulation in 3T3-L1 preadipocytes [38].

#### 4.3.5. *Diaporthe* sp. 3 (**357**–**359**)

The mangrove endophytic fungus *Diaporthe* sp. QYM12 yielded diaporpenoid A (**357**), a novel diterpenoid featuring an unprecedented 5/10/5-fused tricyclic ring system. Compound **357** exhibited potent anti-inflammatory activity, inhibiting NO production in LPS-induced RAW264.7 macrophages with an IC_50_ of 21.5 μM [42].

Separately, chemical investigation of the deep sea-derived fungus *D. longicolla* FS429 afforded two novel diterpenoids, longidiacids A/B (**358**/**359**). Compound **358** inhibited *Mycobacterium tuberculosis* MptpB enzyme activity by 35.4% at 50 μM [100].

#### 4.3.6. *Eutypella* sp. 8 (**360**–**367**)

The Arctic-derived fungus *Eutypella* sp. D-1 produces four pimarane-type diterpenes, **360**–**363**, featuring compound **360**’s rare peroxide bridge structure. Among these, **363** exhibits dual bioactivities: it inhibits LPS-induced nitric oxide release in RAW264.7 macrophages and demonstrates antibacterial effects against *Escherichia coli* and *Staphylococcus aureus* (MIC = 25, 25 μg/mL) [46]. Employing an OSMAC strategy with ethanol induction, three novel pimarane diterpenes were characterized from this fungal strain: eutypellenone F (**364**), libertellenone Y (**365**) with a rare tetrahydrofuran-fused skeleton, and libertellenone Z (**366**). Compound **366** significantly suppressed inflammatory responses at 10 μM (NO inhibition in vitro) and 40 μM (migration inhibition in vivo) [101].

Separately, eutyditerpenoid A (**367**) was isolated from marine *E. scoparia* GZU-4-19Y derived from *Onchidium* sp., representing the first pimarane-type diterpenoid featuring an unprecedented 6/7/6 tricyclic ring system with an anhydride moiety [102].

#### 4.3.7. *Hypoxylon* sp. 2 (**368** and **369**)

Two new diterpenoids, hypoxyterpoids A (**368**) and B (**369**), were isolated from the mangrove-derived fungus *Hypoxylon* sp. Compound **368** demonstrated moderate *α*-glucosidase inhibition with an IC_50_ value of 741.5 µM [103].

#### 4.3.8. *Neocucurbitaria* sp. 15 (**370**–**384**)

Seven novel phomactin diterpenes, neocucurbins A–G (**370**–**376**), were isolated from the deep sea sediment-derived fungus *Neocucurbitaria unguis-hominis* FS685. These compounds represent two distinct structural classes: neocucurbins A–C feature an unprecedented polyoxygenated 5/6/12 or 5/6/13 tricyclic system, while neocucurbins D–G possess a 5/6 bicyclic system formed through macrocyclic ring cleavage, both novel modifications in the phomactin family [104].

Further investigation of this strain yielded densely oxidized phomactins neocucurbols A–H (**377**–**384**), which exhibit two additional novel scaffolds: neocucurbols A–D contain a complex 6/6/5/5/6 polycyclic system with a tetrahydofuran bridge, and neocucurbols E–H feature a 6/8/6 tricyclic system. These findings contrast with the characteristic bicyclo[9.3.1]pentadecane core of previously reported phomactin diterpenes [105].

#### 4.3.9. *Paraconiothyrium* sp. 5 (**385**–**389**)

Five highly cyclized diterpenoids, hawanoids A–E (**385**–**389**), were isolated from the deep sea *Paraconiothyrium hawaiiense* FS482. Compounds **385** and **386** feature a rare tetracyclo [6.6.2.0^2,7^.0^11,15^]cetane skeleton, while **387** and **388** contain a rare 11,14-macrocyclic ether moiety, a novel modification of diterpenoid. All compounds inhibited platelet aggregation induced by the platelet-activating factor, of which **387** and **389** exhibited potent activities with IC_50_ values of 7.1 μM and 8.9 μM [106].

#### 4.3.10. *Penicillium* sp. 9 (**390**–**398**)

Chemical analysis of the organic extract derived from the deep sea fungus *Penicillium thomii* YPGA3 resulted in the isolation of three new labdane-type diterpenoids: 3*β*-hydroxy-agathic acid (**390**), 3*β*-acetoxy-agathic acid (**391**) [107], and penitholabene (**392**) [108]. Notably, penitholabene (**392**) constitutes the first naturally occurring 19-*nor* labdane diterpenoid and exhibited significant *α*-glucosidase inhibitory activity with an IC_50_ value of 282 μM, surpassing that of the positive control acarbose (IC_50_ = 1.33 mM) [108].

From the sea sediment-derived fungus *Penicillium* sp. TJ403-2, three rare cyclopiane diterpenes with a highly fused and strained 6/5/5/5 ring skeleton: 13*β*-hydroxy conidiogenone C, 12*β*-hydroxy conidiogenone C, and 12*β*-hydroxy conidiogenone D (**393**–**395**) were isolated and identified. Compounds **393**–**395** displayed potent anti-inflammatory activity by inhibiting NO production in RAW264.7 cells (IC_50_, 2.19–10.23 μM). Notably, **393** demonstrated particularly potent inhibitory effects with an IC_50_ value of 2.19 μM, significantly lower than that of indomethacin (IC_50_ = 8.76 μM); mechanistic studies using Western blot and immunofluorescence confirmed that its action involves suppression of the NF-*κ*B-activated pathway [109].

Further investigation of the mangrove-associated fungus *P. oxalicum* HLLG-13 yielded two additional cyclopiane diterpenes, conidiogenones J and K (**396** and **397**), from its fermented broth’s EtOAc extract. These compounds weakly inhibited the growth of newly hatched *Helicoverpa armigera* larvae, displaying an IC_50_ value of 200 μg/mL [110].

Characterization of diaporthein C (**398**) from *P. sclerotiorum* GZU-XW03-2 revealed it as only the third example of a pimarane diterpene incorporating a distinctive double bond spanning C_8_ and C_9_ [111].

#### 4.3.11. *Peroneutypa* sp. 1 (**399**)

Bioassay-guided fractionation of the ethyl acetate extract from the marine fungus *Peroneutypa* sp. M16 yielded a new diterpenoid peronepene (**399**) [112].

#### 4.3.12. *Pestalotiopsis* sp. 1 (**400**)

From the mesophotic zone sponge-associated fungus *Pestalotiopsis* sp. NBUF145, researchers isolated pestanoid A (**400**), a structurally rearranged pimarane diterpenoid. This compound demonstrated potent inhibition of osteoclast formation (IC_50_ = 4.2 μM) without cytotoxic effects. Mechanistic studies revealed its dual action in suppressing RANKL-induced osteoclastogenesis through the inhibition of MAPK phosphorylation (ERK1/2, JNK1/2, and p38) and blockade of NF-*κ*B nuclear translocation [113].

#### 4.3.13. *Phoma (Didymella)* sp. 5 (**401**–**405**)

Gene cluster analysis of *Phoma (Didymella)* sp. ATCC 74077 identified the *phm* biosynthetic pathway, enabling the discovery of five new phomactin derivatives: phomactin W; (*S*)-(+)-cembrene A; and phomactin B3, B4, and V1, 4**01**–**405** [114].

#### 4.3.14. *Pleospora* sp. 18 (**406**–**423**)

The mangrove-associated fungus *Pleospora* sp. HNQQJ-1 produced an exceptional series of 18 new isopimarane-type diterpenoids (pleosmaranes A–R, **406**–**423**). These compounds display three distinct structural features: (1) unprecedented 20-*nor-*isopimarane skeletons with aromatic B rings (**406**–**414**), (2) novel 2-oxabicyclo[2.2.2]octane units (**420**–**422**), and (3) a rare 2-oxabicyclo[3.2.1]octane system (**423**). Notably, compounds **413** and **417** showed moderate inhibition of LPS-induced NO production (IC_50_ = 19 and 25 μM, respectively) [115].

#### 4.3.15. *Stachybotrys* sp. 15 (**424**–**438**)

The coral-associated toxigenic fungus *Stachybotrys chartarum* afforded five previously unidentified atranones (atranones V–Z, **424**–**428**) and three novel dolabellane-type diterpenoids (stachatranones D–F, **429**–**431**). Among these, compound **431** demonstrated remarkable dose-dependent cardioprotective effects, significantly reducing cold ischemic (CI) injury in cardiomyocytes. Mechanistic studies revealed its ability to inhibit PI3K/AKT pathway-mediated apoptosis to protect against oxidative stress-induced CI injury [116].

Subsequent investigation of this strain identified seven additional diterpenoids (stachybatranones A–F, **432**a/**433**b and **434**–**438**) from this strain. These compounds exhibited two distinct structural classes: (1) rare C-alkylated dolabellanes (**434**–**438**), characterized by a unique five-membered hemiketal ring and a *γ*-butyrolactone moiety fused to an 11-membered carbocyclic core, and (2) unprecedented fused atranone (**432**a/**433**b), representing the first 5–11–6-fused atranone scaffold, incorporating a 2,3-butanediol unit. Biological assessment confirmed that multiple compounds (**432**a/**433**b and **436**) provided dose-dependent protection against CI injury, with **432**a/**433**b specifically maintaining PI3K/AKT phosphorylation. These findings highlight the potential of fungal diterpenoids as novel cardioprotective agents for transplant medicine [117].

#### 4.3.16. *Talaromyces* sp. 25 (**439**–**463**)

Initial isolation of deep sea anemone endophyte *Talaromyces scorteus* AS-242 yielded eight new diterpenoid acids (**439**–**446**), talascortenes A–G, and 5*α*,9*β*-dihydroxyisocupressic acid, exhibiting four distinct carbon skeletons. All compounds displayed broad-spectrum antimicrobial activity against human, aquatic, and plant pathogens (MIC range: 1–32 μg/mL) [118]. Further investigation identified four new diterpenoids (talascortenes H–K, **447**–**450**), including two 19-*nor-*diterpenoids (**447** and **448**) and two diterpenoid acids (**449** and **450**), from the same strain. Compound **447** exhibited inhibitory activity against *Curvularia spicifera* (MIC, 4 μg/mL). Compound **448** exhibited superior efficacy against *C. spicifera* (MIC = 1 μg/mL). Compound **449** demonstrated potent activity against pathogenic fungi *Fusarium oxysporum* and *Penicillium digitatum* (MIC, 2, 2 μg/mL) and bacteria *Micrococcus luteus* (MIC, 4 μg/mL), while **450** showed significant fungicidal effects against *Bipolaris sorokiniana* (MIC, 2 μg/mL) and *F. proliferatum* (MIC, 4 μg/mL) [119].

Heterologous expression of the silenced *labd* cluster from marine fungus *Talaromyces* sp. HDN151403 led to the discovery of five novel labdane diterpenes, talarobicins A–E (**451**–**455**), representing four skeleton types. Talarobicin B (**452**) is the first 3,18-dinor-2,3:4,18-*diseco-*labdane diterpene, resulting from C_2_ to C_3_ bond cleavage and decarboxylation at C_3_ and C_18_, and serves as a key biosynthetic intermediate for penioxalicin [120].

Three 3-*nor-*labdane diterpenes, talaroterpenoids A–C (**456**–**458**), were isolated from marine *T. aurantiacus.* Compounds **456** and **457** feature an unusual 6,20-*γ*-lactone-bridged scaffold. Talaroterpenoid C (**458**) exhibited moderate antifungal activity against *Alternaria alternata* and *Pestalotiopsis theae* (MIC = 50 μg/mL) [121].

From mangrove endophytic *Talaromyces* sp. JNQQJ-4, five new diterpenes were obtained: talaroacids A–D (**459**–**462**), featuring a 1,2,3,4,4a,5,6,8a-octalin skeleton, and an isopimarane-type, talaromarane A (**463**). Compound **463** contains a rare 2-oxabicyclo[3.2.1]octane moiety. Compounds **459**, **460**, **462**, and **463** showed significant anti-inflammatory activities (IC_50_ = 4.59–21.60 μM) [122].

#### 4.3.17. *Trichoderma* sp. 15 (**464**–**478**)

Starfish-derived fungus *Trichoderma erinaceum* F1–1 produced harziandione A (**464**) on L-phenylalanine-deficient GPY medium [93].

Chemical epigenetic manipulation (10 µM of sodium butyrate) of coral-derived *T. harzianum* (XS-20090075) induced the production of harziane diterpenoid, harzianolic acid A (**465**), and the first chlorinated cleistanthane diterpenoid, harzianone E (**466**), with only **466** exhibiting weak activity against *P. angustum* [92].

Deep sea sediment-derived *Trichoderma* sp. SCSIOW21 yielded five undescribed harziane-type derivatives, harzianols K–O (**467**–**471**) [123], and two new aminoglycoside diterpenes, harzianosides A and B (**472** and **473**) [90].

The marine alga-derived *T. asperelloides* RR-dl-6-11 afforded a new proharziane and two new harziane derivatives (**474**–**476**), alongside structurally unique *seco-*harziane **477**. Compounds **474**–**477** inhibited four marine phytoplankton species (IC_50_ = 18–47 μg/mL) [124]. In a separate study, the marine alga epiphytic *Trichoderma* sp. Z43 was found to produce harziaketal A (**478**), which represents the first reported harziane-type diterpene featuring a hemiketal moiety on its characteristic four-membered ring. This compound exhibited moderate phytoplankton growth inhibition (IC_50_, 14–48 μg/mL) [125].

### 4.4. Sesterterpenes 17 Compounds (**479**–**495**)

**Figure 22:**
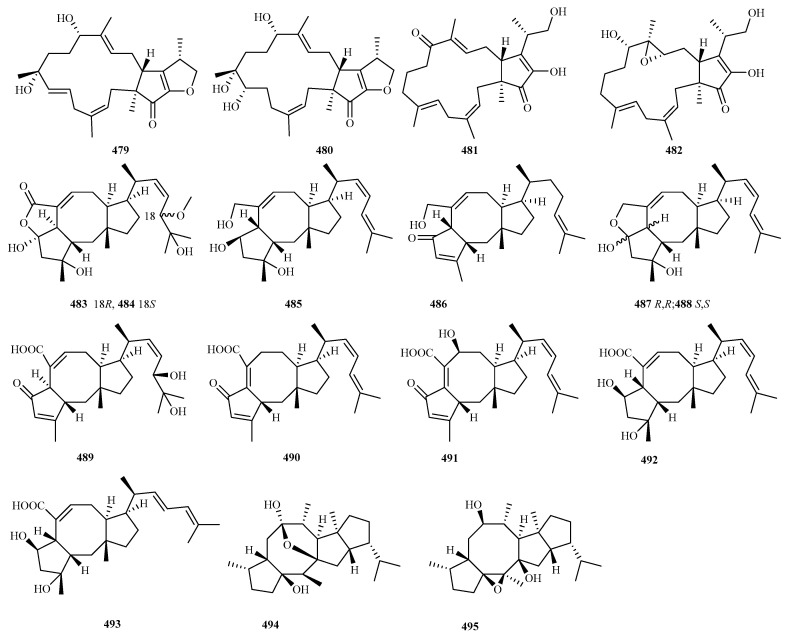
Chemical structures of sesterterpenes (**479**–**495)**.

As of the end of 2024, a total of 74 new sesterterpenoids had been identified from marine fungi, with ophiobolin-type sesterterpenoids representing the predominant structural class [8,9,10]. Seventeen novel compounds (**479**–**495** in Figure 22), including eleven ophiobolin derivatives, were discovered across six articles within the preceding five year. *Aspergillus* spp. served as the primary source of these fungal sesterterpenoids, yielding 15 compounds. These sesterterpenes consistently exhibited anti-inflammatory and antimicrobial activities.

The fungus *Arthrinium* sp. SCSIO41221, isolated from mangrove sediment, yielded four new sesterterpenes, arthproliferins A–D (**479**–**482**). Among these, arthproliferin B (**480**) exhibited weak cytotoxic effects against U87MG glima cells [126].

Chemical investigation of *Aspergillus* sp. RR-YLW-12, an epiphyte on the red alga *Rhodomela confervoides*, led to the isolation of three new ophiobolin-type sesterterpenoids: the C_18_ epimers 18,19-dihydro-18-methoxy-19-hydroxyophiobolin P (**483** and **484**) [28] and 21-deoxo-21-hydroxyophiobolin U (**485**) [29]. Compound **485** demonstrated significant growth inhibition against four marine microalgae (*Prorocentrum donghaiense*, *Heterosigma akashiwo*, *P. micans*, and *H. circularisquama*), with IC_50_ values ranging from 6.3 to 12.9 μg/mL [29].

Ten new sesterterpenoids (**486**–**495**) were discovered from three distinct deep sea-derived fungi. *Aspergillus insuetus* SD-512 (cold seep sediment, 1331 m) produced three new ophiobolin derivatives: (6*R*)-16,17,21,21-*O*-tetrahydroophiobolin G (**486**), (6*R*)-16,17-dihydroophiobolin H (**487**), and (5*S*,6*S*)-16,17-dihydroophiobolin H (**488**). Compound **488** displayed broad-spectrum antibacterial activity (MIC, 4–32 μg/mL) [127]. *Aspergillus* sp. WHU0154 yielded five new ophiobolins (**489**–**493**): 18,19-dihydro-18,19-dihydroxyasperophiobolin E, ∆^16,17^-8-dehydroxyophiobolin D, ∆^16,17^-ophiobolin D, asperophiobolin L, and (16*E*)-asperophiobolin L. Compound **492** significantly inhibited NO production in LPS-stimulated RAW264.7 macrophages [128]. Chemical analysis of *Chaetomium globosum* SD-347 afforded sesterchaetins A and B (**494** and **495**), characterized by a rare 5/8/6/5 tetracyclic ring system. These compounds exhibited selective antimicrobial activity against human, aquatic, and plant pathogens (MIC, 8–32 μg/mL); structural analysis suggests the hemiketal moiety influences the antibacterial effects, while the unique scaffold governs antifungal activity [129].

### 4.5. Triterpenes 17 Compounds (**496**–**512**)

**Figure 23:**
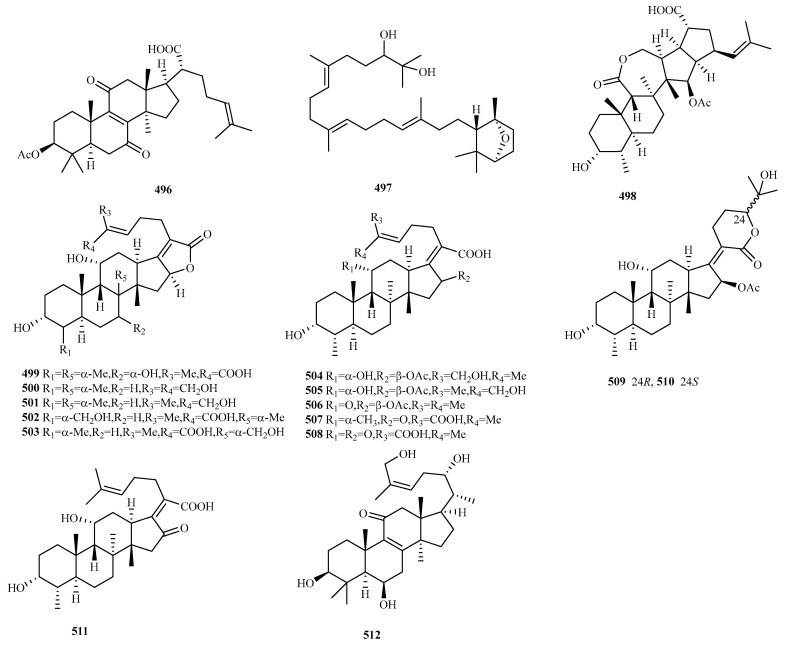
Chemical structures of triterpenes (**496**–**512**).

Triterpenes (excluding steroids) are exceptionally rare in marine fungi. As of the end of 2024, only 28 novel triterpenes had been discovered from this source [8,9,10]. Significantly, 17 of these new compounds (**496**–**512**, Figure 23) were identified across five articles within the preceding five years, indicating substantial potential for future discoveries of this compound class.

Shellfish-derived *Ceriporia lacerata* CD7-5 yielded a new lanostane-type triterpenoid **496** (3*β*-acetoxy-7,11-dioxolanosta-8,24-dien-21-oic acid). Compound **496** demonstrated significant inhibitory activity against three microalgal species (IC_50_ = 5.7–26.3 μg/mL) and exhibited 52.9% inhibitory against zooplankton *Artemia salina* at 90 μg/mL [130].

Deep sea sediment-derived *Phomopsis lithocarpus* FS508 produced lithocarin D (**497**), a novel triterpenoid isolated from its broth extract [131].

Soft coral-sourced *Simplicillium* sp. SCSIO 41513 afforded eleven unprecedented fusidane-type nortriterpenoids, simplifusidic acids A–K (**498**–**508**). Among these, compound **498** features a novel 6/6/7/5/5 pentacyclic fusidane skeleton, and compounds **499–505**, **507**, and **508** display unique diversely substituted side chains at C17. Bioassays showed compound **506** and **505** had potent antibacterial activity against *Staphylococcus aureus* (MIC = 0.078, 2.5 μg/mL), and SAR revealed that the chemical structure of fusidic acid was optimal for its antibacterial activity, and the C21 carboxylic acid group was indispensable for its antibacterial activity [132].

The Arctic marine-derived fungus *S. lamellicola* yielded three additional fusidane-type nortriterpenoids: simplifusinolide A (**509**), 24-*epi*-simplifusinolide A (**510**), and simplifusidic acid L (**511**). Compounds **510** and **511** exhibit potential as alternative agents for benign prostatic hyperplasia treatment, acting via androgen/androgen receptor signaling pathway modulation, and SAR indicated that the C_16_ acetyl group could be indispensable for their bioactivities [133].

From the sea-anemone-associated endophyte *Talaromyces scorteus* AS-242, researchers isolated one new triterpenoid talascortene L (**512**) [119].

## 5. Conclusions and Perspectives

This review synthesizes the chemical structural diversity and bioactive potential of terpenoids derived from marine fungi during 2020–2024, marking a transformative era in marine natural product discovery. A remarkable 512 new terpenes were isolated from 104 fungal strains across 34 genera, nearly equaling the cumulative total reported prior to 2020 (586 compounds). The structural distribution is dominated by sesquiterpenes (345 compounds, 68%) and diterpenes (128 compounds, 25%), with the major producing fungal genera including *Trichoderma* (87 compounds, 17%), *Aspergillus* (84, 16%), *Eutypella* (71, 14%), and *Penicillium* (59, 12%). This observation aligns with the consensus in reviews on plant-derived endophytic fungi, where sesquiterpenoids constitute the predominant class of newly reported terpenes, with *Trichoderma*, *Aspergillus*, and *Penicillium* being the major genera of origin. However, comparative analysis reveals that marine fungi exhibit a significantly higher yield of novel terpenoid compounds [4,5]. For the first time, fungi directly isolated from marine environments (51%, 260 of sources) surpassed those associated with marine organisms (47%, 243). Key marine sources included deep sea sediments (26% of strains), marine invertebrate symbioses (21%), algal associations (14%), and mangrove ecosystems (12%). Fungi from extreme environments (e.g., deep sea/hydrothermal vents and polar regions) exhibit exceptional terpene structural diversity. Approximately 57% of these terpenoids (266 compounds) demonstrated broad-spectrum bioactivities across 23 categories, documented in 305 distinct reports: anti-inflammatory (88 reports, 29%), antibacterial (51 reports, 17%), antimicroalgal (51 reports, 17%), antifungal (46 reports, 15%), and cytotoxic (19 reports, 6%) activities. Notably, marine fungal terpenes demonstrating high potency warrant prioritization in drug screening. Representative compounds with significant bioactivities include anti-inflammatory (**123** [47], paraconulone D **248** [70], and 13*β*-hydroxy conidiogenone C **393** [109], IC_50_ = 2.2 μM or ex vivo); antibacterial (chermesiterpenoids B–C **214**–**215** [59], pseuboyene I **228** [64], and simplifusidic acid I **506** [132], MIC = 0.078–2 μg/mL); antimicroalgal (trichobisabolins Q–R **291**–**292** and cadin-4-en-11-ol **301**, IC_50_ = 0.5–1.0 μg/mL [81]); antifungal (pseuboyenes C–E **222**–**224** [64] and talascortene I **448** [119], MIC = 0.5–1 μg/mL); cytotoxic (insulicolides F–G **71**–**72** [32] and copteremophilanes H **176** [52], IC_50_ = 2.3–3.2 µM); antiviral (acremosides A **7**, EC_50_ = 4.8 μM, ref. [17]); ferroptosis inhibition (12-hydroxyengyodontiumone I **50**, IC_50_ 2.1 = µM, ref. [25]); AChE inhibition (bisabolanoic acid A **94**, IC_50_ = 2.2 μM, ref. [40]); antiangiogenic (eutypene I **164**, ex vivo, ref. [50]); platelet aggregation inhibition (hawanoid C **387**, IC_50_ = 7.1 μM, ref. [106]); osteoclastogenesis inhibition (pestanoid A **400**, IC_50_ = 4.2 μM, ref. [113]); cardioprotective (stachatranone F **431** [116]; and stachybatranones A **432**/**433**, ref. [117]).

The development of marine fungal terpenoids as viable drug candidates faces multifaceted challenges, primarily stemming from low production yields due to scale-up barriers; methodological limitations in bioassays. incomplete lead compound studies (encompassing structural optimization, ADMET profiling, and pharmacokinetics); and insufficient mechanistic understanding of pharmacological activities. To address these gaps, a system-driven drug discovery framework is proposed, emphasizing pharmacophore mapping to identify critical bioactive motifs; standardized biological testing protocols to ensure reproducibility; and comprehensive research methodologies integrating genome mining, synthetic biology, and multi-omics approaches (e.g., metabolomics and genomics) for system-level exploration. A paradigm shift has been exemplified by recent advances in marine fungal cyclopiane diterpenes, where total synthesis and mechanistic dissection—particularly the identification of direct anti-inflammatory targets—have established a robust model for marine-derived drug development [134]. The integration of advanced metabolomics and genomics is poised to accelerate more discoveries from the chemically rich yet underexplored marine fungal repertoire [135,136,137]. Targeted bioprospecting and interdisciplinary approaches are critical to harness this resource for addressing unmet medical needs.

## Data Availability

No new data were created or analyzed in this study. Data sharing is not applicable to this article.

## Supplementary Materials

The following supporting information can be downloaded at: https://www.mdpi.com/article/10.3390/md23080300/s1: Figure S1: Maximum-likelihood phylogenetic tree of representative marine fungi based on ITS sequences.
