# Supplementary material for: Structural Diversity and Bioactivities of Marine Fungal Terpenoids (2020–2024)
Source: Mar Drugs. 2025 Jul 27;23(8):300. doi: 10.3390/md23080300 (PMC12387476; doi:10.3390/md23080300)
Supplement: Supplementary file 1 [file marinedrugs-23-00300-s001.zip › marinedrugs-3768092-supplementary.pdf]

Supplementary Materials

Structural Diversity and Bioactivities of Marine Fungal Terpenoids (2020–2024)

Minghua Jiang [1], Senhua Chen [2,3,4,*], Zhibin Zhang [5], Yiwen Xiao [1], Du Zhu [1,5,*] and Lan Liu [2,3,4,]

[1] Key Laboratory of Natural Microbial Medicine Research of Jiangxi Province, College of Life Sciences, Jiangxi Science and Technology Normal University, Nanchang 330013, China; jiangmh23@mail2.sysu.edu.cn (M.J.); xyw1152858687@163.com (Y.X.);

[2] School of Marine Sciences, Sun Yat–sen University, Zhuhai 519000, China; cesllan@mail.sysu.edu.cn(L.L.).

[3] State Key Laboratory of Environmental Adaptability for Industrial Products, Zhuhai 519082, China;

[4] Southern Marine Sciences and Engineering Guangdong Laboratory (Zhuhai), Zhuhai 519000, China;

[5] Jiangxi Province Key Laboratory of Biodiversity Conservation and Bioresource Utilization, College of Life Sciences, Jiangxi Normal University, Nanchang 330022, China; zzbbio@jxnu.edu.cn (Z.Z.)

* Correspondence: chensenh@mail.sysu.edu.cn (S.C.); zhudu@jxstnu.edu.cn (D.Z.).

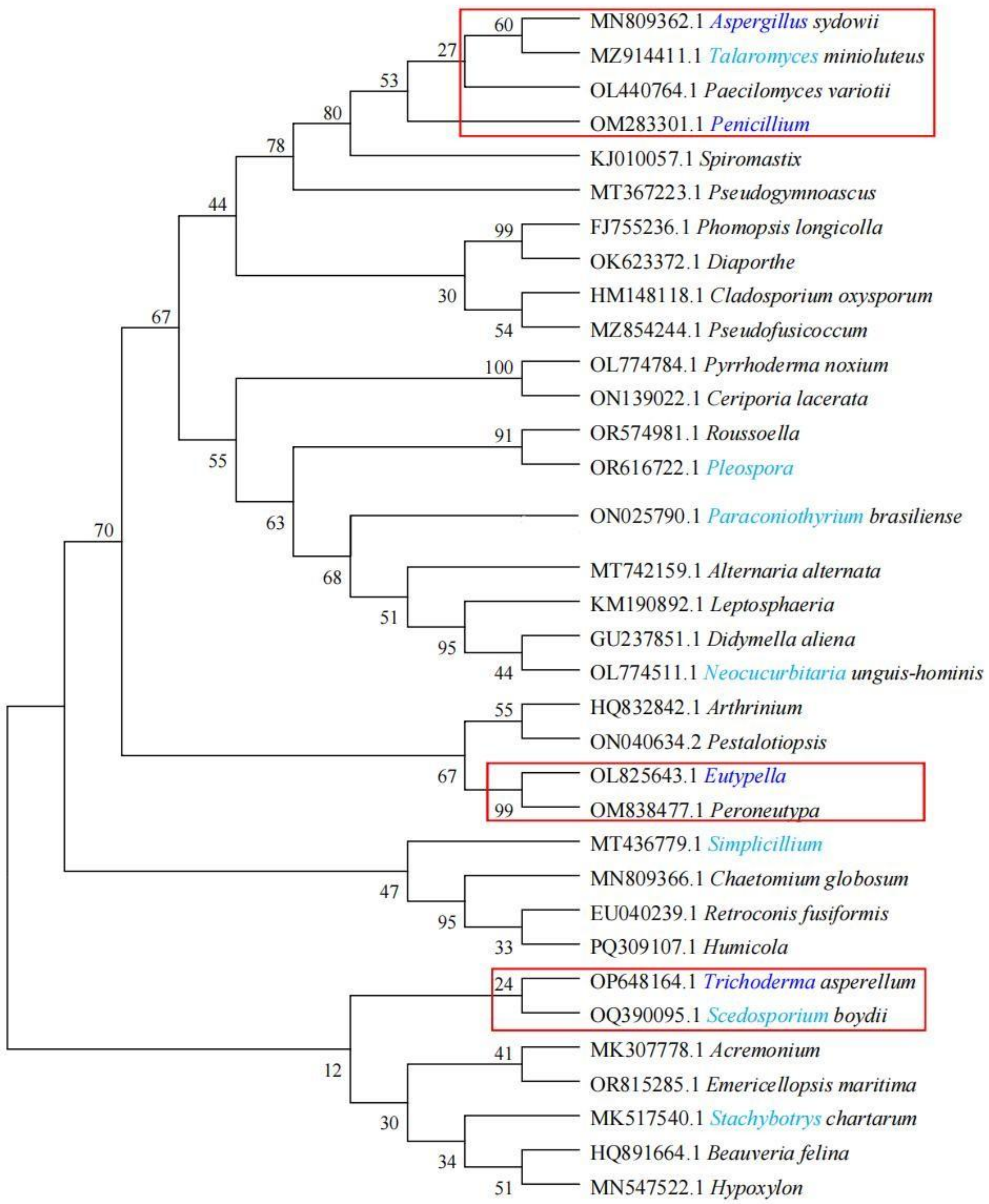

Figure S1： Maximum-likelihood phylogenetic tree of representative marine fungi based on ITS sequences. Branches highlighted in red indicate genera with demonstrated high terpenoid productivity (*Aspergillus, Talaromyces, Penicillium, Trichoderma, Scedosporium,Eutypella*) or predicted potential (*Paecilomyces, Peroneutypa*).
